# Notch-Mediated Tumor-Stroma-Inflammation Networks Promote Invasive Properties and CXCL8 Expression in Triple-Negative Breast Cancer

**DOI:** 10.3389/fimmu.2019.00804

**Published:** 2019-04-24

**Authors:** Yulia Liubomirski, Shalom Lerrer, Tsipi Meshel, Dina Morein, Linor Rubinstein-Achiasaf, David Sprinzak, Stefan Wiemann, Cindy Körner, Marcelo Ehrlich, Adit Ben-Baruch

**Affiliations:** ^1^School of Molecular Cell Biology and Biotechnology, George S. Wise Faculty of Life Sciences, Tel Aviv University, Tel Aviv, Israel; ^2^School of Neurobiology, Biochemistry & Biophysics, George S. Wise Faculty of Life Sciences, Tel Aviv University, Tel Aviv, Israel; ^3^Division of Molecular Genome Analysis, German Cancer Research Center (DKFZ), Heidelberg, Germany

**Keywords:** cancer-associated fibroblasts, CXCL8, interleukin 1β, mesenchymal stem cells, notch1, p65, triple-negative breast cancer, tumor necrosis factor α

## Abstract

Stromal cells and pro-inflammatory cytokines play key roles in promoting the aggressiveness of triple-negative breast cancers (TNBC; Basal/Basal-like). In our previous study we demonstrated that stimulation of TNBC and mesenchymal stem cells (MSCs) co-cultures by the pro-inflammatory cytokine tumor necrosis factor α (TNFα) has led to increased metastasis-related properties *in vitro* and *in vivo*. In this context, elevated release of the pro-metastatic chemokines CXCL8 (IL-8) and CCL5 (RANTES) was noted in TNFα- and interleukin-1β (IL-1β)-stimulated TNBC:MSC co-cultures; the process was partly (CXCL8) and entirely (CCL5) dependent on physical contacts between the two cell types. Here, we demonstrate that DAPT, inhibitor of γ-secretase that participates in activation of Notch receptors, inhibited the migration and invasion of TNBC cells that were grown in “Contact” co-cultures with MSCs or with patient-derived cancer-associated fibroblasts (CAFs), in the presence of TNFα. DAPT also inhibited the contact-dependent induction of CXCL8, but not of CCL5, in TNFα- and IL-1β-stimulated TNBC:MSC/CAF co-cultures; some level of heterogeneity between the responses of different TNBC cell lines was noted, with MDA-MB-231:MSC/CAF co-cultures being the most sensitive to DAPT. Patient dataset studies comparing basal tumors to luminal-A tumors, and mRNA analyses of Notch receptors in TNBC and luminal-A cells pointed at Notch1 as possible mediator of CXCL8 increase in TNFα-stimulated TNBC:stroma “Contact” co-cultures. Accordingly, down-regulation of Notch1 in TNBC cells by siRNA has substantially reduced the contact-dependent elevation in CXCL8 in TNFα- and also in IL-1β-stimulated TNBC:MSC “Contact” co-cultures. Then, studies in which CXCL8 or p65 (NF-κB pathway) were down-regulated (siRNAs; CRISPR/Cas9) in TNBC cells and/or MSCs, indicated that upon TNFα stimulation of “Contact” co-cultures, p65 was activated and led to CXCL8 production mainly in TNBC cells. Moreover, our findings indicated that when tumor cells interacted with stromal cells in the presence of pro-inflammatory stimuli, TNFα-induced p65 activation has led to elevated Notch1 expression and activation, which then gave rise to elevated production of CXCL8. Overall, tumor:stroma interactions set the stage for Notch1 activation by pro-inflammatory signals, leading to CXCL8 induction and consequently to pro-metastatic activities. These observations may have important clinical implications in designing novel therapy combinations in TNBC.

## Introduction

The triple-negative subtype of breast cancer (TNBC), which in gene signature studies is often used as a surrogate for the “Basal/Basal-like” subgroup (e.g., in PAM50 analyses), accounts for ~15% of breast cancers. TNBC cells are negative for the expression of type α estrogen receptors, progesterone receptors or amplified HER2; thus, TNBC tumors do not respond to receptor-targeted therapies, and following chemotherapy they are most likely to recur (1–3). These clinical parameters emphasize the ultimate need for improved understanding of the mechanisms leading to tumor progression in this aggressive subtype of disease.

Key roles in regulating tumor progression in TNBC are attributed to elements of their surrounding tumor microenvironment (TME) (4, 5). We and others have investigated the interactions of TNBC cells with TME elements which promote tumor development and metastasis-related functions in TNBC: (1) Stromal cells such as mesenchymal stem cells (MSCs) and cancer-associated fibroblasts (CAFs) that enhance TNBC progression by releasing pro-angiogenic factors and additional tumor-promoting mediators (6–15); (2) Pro-inflammatory cytokines, particularly tumor necrosis factor α (TNFα) and interleukin 1β (IL-1β) - or their signaling-related components which are expressed in TNBC tumors - and promote the aggressiveness profile of TNBC cells (16–25).

Because information is lacking on the outcomes of TNBC interactions with stromal cells in the context of pro-inflammatory signals, we have studied in a companion research (26) the effects of tumor-stroma-inflammation networks on pro-metastatic processes in TNBC. We demonstrated that TNFα stimulation of TNBC:MSC “Contact” co-cultures has led to enhanced migration and invasion of TNBC cells, to increased angiogenesis and to higher metastatic potential of the tumor cells *in vivo*. Moreover, TNFα- and IL-1β-stimulated TNBC:MSC/CAF co-cultures released elevated levels of CXCL8 and CCL5, identified as pro-metastatic chemokines in TNBC (27–36). The release of CXCL8 by TNFα- and IL-1β-stimulated TNBC cells grown with MSCs/CAFs was partly dependent on the exchange of soluble factors between the TNBC cells and the stromal cells, but also required direct physical contacts between these two cell types. In contrast, induction of CCL5 in TNFα- and IL-1β-stimulated TNBC:MSC co-cultures was entirely dependent on cell-to-cell contacts. Of importance, CXCL8 induction in the context of TNFα-stimulated TNBC:MSC “Contact” co-cultures was significantly involved in mediating the increased metastasis-related phenotypes of TNBC cells; this included induction of angiogenesis, as well as of the migratory and invasive properties of tumor cells.

These findings provided novel insights to processes controlling TNBC aggressiveness, and have led us to investigate in the current study the mechanisms involved in such tumor-stroma-inflammation networks. Here, we were specifically out to unravel the regulation of processes that necessitated physical contacts between the tumor cells and stromal cells. Along these lines, we focused on the potential roles of the Notch pathway in controlling the tumor-stroma-inflammation networks we have identified in TNBC (26).

The Notch pathway regulates differentiation, proliferation, and cell death through direct cell-to-cell signaling (37–40). Following receptor-ligand interactions, a series of proteolytic cleavages in the Notch receptor lead to the γ-secretase-dependent release of the Notch intracellular domain (NICD); NICD then translocates to the nucleus, where it forms an activator complex that regulates the transcription of target genes controlling various regulatory and functional programs (37–40). In breast cancer, particularly in TNBC, increasing evidence indicates that Notch family members are ultimate contributors to cancer stem cell maintenance, invasion, angiogenesis and recurrence (41–45). However, the Notch pathway was not explored so far for its involvement in regulating inflammation-driven TNBC-stroma interactions.

Thus, in this study we investigated the roles of Notch receptors in regulating such cross-talks using the research system we have described in our accompanying study (26). We now demonstrate that the Notch pathway is a prime regulator of tumor cell invasiveness in the tumor-stroma-inflammation setting. Also, our findings indicate that NF-κB-induced Notch1 activation is a key regulator of inflammation-driven TNBC-stromal contacts that lead to elevated release of the pro-metastatic chemokine CXCL8; as we have shown before, CXCL8 then contributed to elevated angiogenesis, tumor cell migration and tumor cell invasion in the tumor-stroma-inflammation network in TNBC (26). Together, our findings point at complex control mechanisms that are governed by the NF-κB and Notch pathways in the setting of TNBC-stroma-inflammation triage that promotes TNBC progression, and may have clinical implications.

## Materials and Methods

### Breast Tumor Cell Lines and Stromal Cells

The TNBC human MDA-MB-231, MDA-MB-468 and BT-549 cells (ATCC), and human luminal-A MCF-7 cells (ATCC) were grown as previously described (26). Human bone marrow-derived MSCs from three different healthy donors were purchased from Lonza (#PT-2501; Walkersville, MD) and were grown for up to 10 passages, as previously described (26). CAFs that were isolated from patients' breast tumors (from a primary tumor in ELISA studies, and from a lung metastasis in tumor cell invasion studies), were immortalized and grown as described in (6) (Kindly provided by Dr. Bar, Sheba Medical Center, Ramat Gan, Israel).

### TNFα and IL-1β Concentrations Used in Different Analyses

Titration analyses of cytokine stimulation were performed as described in the accompanying study (26). To follow up on those studies, recombinant human (rh) TNFα (#300-01A, PeproTech, Rocky Hill, NJ) and rhIL-1β (#200-01B, PeproTech) were used in the following concentrations: MDA-MB-231 cells, MCF-7 cells, MSCs and CAFs: TNFα 10 ng/ml, IL-1β 350 pg/ml; MDA-MB-468 cells: TNFα 50 ng/ml, IL-1β 500 pg/ml; BT-549 cells: TNFα 25 ng/ml, IL-1β 350 pg/ml.

### Tumor Cell Migration and Invasion

In migration assays, mCherry-expressing MDA-MB-231 cells were added to the upper part of transwells (8-μm pore membranes; #3422, Corning, NY) together with MSCs (ratio 10:1). The cells were stimulated by TNFα, and were treated by DAPT [10 μM; (N-[N-(3,5-difluorophenacetyl)-l-alanyl]-S-phenylglycine t-butyl ester]; #565770; Calbiochem, Merck, Darmstadt, Germany) or by its vehicle control (Dimethylsulfoxide, DMSO; #D5879; Sigma-Aldrich, Merck KGaA, Darmstadt, Germany). Migration assays toward medium with 10% FBS were performed for 12 h. Then, cells at the upper side of the membranes were removed, the membranes were fixed in ice-cold methanol and stained with Hemacolor (#1.11661; Merck, Darmstadt, Germany). Photos of multiple high power fields were taken in bright fields and in fluorescent fields at ×100 magnification. After verifying that the transmigrating cells expressed mCherry (and thus were tumor cells), the cells at the lower side of the membranes were counted in multiple Hemacolor fields.

In invasion assays, 3D multicellular spheroids of defined size and cell number were formed by co-culturing of mCherry-expressing MDA-MB-231 and MSCs/CAFs (ratio 10:1) in hanging drops [as in Korff et al. (46), with minor modifications] for 72 h. These experiments were performed in the presence of DAPT (10 μM) or control DMSO. The spheroids were then embedded in matrigel (9–10.5 mg/ml; #356234, Corning, Bedford, MA) and stimulated by TNFα (10 ng/ml) with fresh DAPT (10 μM) or control DMSO. Invasion of mCherry-tumor cells out of spheroids that were formed with MSCs was determined after 48 h or 96 h with CAFs. Multiple MDA-MB-231:MSC spheroids were photographed in fluorescent fields at ×40 magnification, and the invaded areas were determined by mCherry signals of cells that invaded out of spheroid cores, quantified by ImageJ. In parallel, many MDA-MB-231:CAF spheroids were photographed in fluorescent fields and in bright fields at ×100 magnification. Quantification by ImageJ was performed in fluorescent fields at × 40magnification.

### Cell Stimulation for ELISA Assays

TNBC cells were grown together with MSCs/CAFs (10:1 ratio) in “Contact” conditions (in which the two cell types could form physical contacts) in 6-well plates (#3516, Corning, Kennebunk, ME); in parallel, similar cell concentrations were used to generate “Transwell” conditions (in which the two cell types could only exchange soluble materials between them) in 6-well plates, with an insert of 0.4 μm permeable polycarbonate membrane (#3412, Corning). In the studies demonstrated in Supplementary Figures 2A,B, separate cultures of TNBC cells and MSCs were also included, grown individually in the same cell numbers as in co-cultures. Co-cultured cells, and individual cell types (when appropriate) were grown in media containing 10% FBS for 12 h and were then stimulated by TNFα or IL-1β in media containing 0.5% FBS for 7 h. Following removal of cytokine stimulation, cytokine-free media supplemented with 0.5% FBS were added for additional 60 h. Then, conditioned media (CM) were removed and cleared by centrifugation, and CXCL8 and CCL5 extracellular levels were determined by ELISA, using standard curves at the linear range of absorbance. To this end, rhCXCL8 (#200-8M, PeproTech), and antibodies (Abs) to CXCL8 were used (Coating Abs: #500-P28. Detecting Abs: #500-P28Bt; PeproTech). In parallel, CCL5 levels were detected by using rhCCL5 (#300-06; PeproTech) and Abs to CCL5 (Coating Abs: #500-M75; PeproTech. Detecting Abs: #BAF278; R&D Systems, Inc., Minneapolis, MN). Following the addition of HRP-conjugated Streptavidin (#016-030-084; Jackson Immunoresearch laboratories, PA) and substrate TMB/E solution (#ES001; Millipore, Temecula, CA), the reaction was stopped by addition of 0.18 M H_2_SO_4_. Absorbance was measured at 450 nm.

When indicated, cell cultures were treated by DAPT (10 μM) or by its vehicle control (DMSO); Down-regulation of CXCL8 and NOTCH1 expression by siRNA was introduced in other experiments, as detailed below. CM that were collected from such co-cultures were analyzed for CXCL8 and/or CCL5 expression by ELISA, as detailed above.

### Analyses of Patient Datasets

The cancer genome atlas (TCGA) dataset of breast cancer patient (47) was used for RNAseq-based gene expression analyses. The TCGA dataset contained samples of 821 patients: Basal (often overlapping the term TNBC): 141 patients; Luminal-A: 421 patients; Luminal-B: 192 patients; HER2+: 67 patients. The PAM50 annotation file provided within the dataset was used to define disease subtypes. Log2-transformed expression values of NOTCH1, NOTCH2, NOTCH3 and NOTCH4 by subtypes, were presented as boxplots. Statistical analyses were performed based on Shapiro-Wilk normality test, for each gene by subtype. Kruskal-Wallis test with correction for multiple comparisons using the Benjamini-Hochberg procedure controlling the false discovery rate (FDR), was used for comparison of expression levels between the different subtypes. Log2-transformed co-expression levels of NOTCH1 or NOTCH2 with genes of interest were determined in basal patients and in luminal-A patients, and were outlined as scatter plots. In NOTCH1 studies, the centroid of the scatter plot (determined by the average values of NOTCH1 and of the second analyzed gene) in luminal-A patients was used to set rectangles demonstrating the shift in basal patients.

### Quantitative Real-Time PCR Analyses

MDA-MB-231 and MCF-7 cells were co-cultured with MSCs (10:1 ratio) under “Contact” conditions; in parallel, when appropriate, each of the cell types was grown alone. Then, the co-cultures/cells were stimulated by TNFα or IL-1β for 7 h in media containing 0.5% FBS. Similar procedures were performed using MDA-MB-231 cells that were subjected to gene down-regulation by siRNA and/or CRISPR/Cas9, as detailed below. Total RNA was isolated using the EZ-RNA kit (#20-400; Biological Industries, Beit Ha'emek, Israel) for quantitative real-time PCR (qRT-PCR) analyses. The M-MLV reverse transcriptase (#AM2044; Ambion, Austin, TX or #95047; Quantabio, Beverly, MA) was used to generate first-strand cDNA from RNA samples. cDNA targets were quantified on Rotor Gene 6000 (Corbett Life Science, Concorde, NSW, Australia) or on CFX Connect real time PCR Detection System (Bio-Rad, Hercules, CA). To detect transcripts, absolute Blue qPCR SYBR Green ROX mix (#AB-4163/A; Thermo Fisher Scientific, Waltham, MA) was used, according to manufacturer's instructions. The housekeeping gene GAPDH was used for data normalization. For each primer set, dissociation curves indicated a single product and “no-template” controls were negative after 40–45 cycles used for analysis. Analyses were performed by standard curves, within the linear range of quantification. The sequences of the primers are provided in Supplementary Table 1.

### Western Blot Analyses

MDA-MB-231:MSC “Contact” co-cultures (ratio 10:1), or each of the cell types alone (when appropriate) were stimulated for 15 min or 7 h by TNFα, IL-1β or vehicle control, in media containing 0.5% FBS. Similar procedures were taken when the co-cultures were subjected to gene down-regulation by siRNA or CRISPR/Cas9, as detailed below. Following lysis in RIPA buffer, conventional Western blot (WB) procedures were taken. To detect p65 expression and activation, the following Abs were used: Total (T)-p65: #8242 [Cell Signaling Technology (CST), Danvers, MA]; Phosphorylated (P)-p65: #3033 (CST). To detect Notch1 expression and activation, the following Abs were used: Full length Notch1: #3608 (CST); Notch1 intracellular domain (N1-ICD; N1-ICD appeared as single band or two bands, probably due to technical reasons): #4147, reacting specifically with cleaved Notch 1 (directed to Val1744; CST). GAPDH (#ab9485, Abcam, Cambridge, UK), served as a loading control. The membranes were reacted with streptavidin-horseradish peroxidase (HRP)-conjugated goat anti-rabbit IgG (#111-035-003) or HRP-conjugated goat anti-mouse IgG (#115-035-071), as appropriate (both from Jackson ImmunoResearch Laboratories). The membranes were subjected to enhanced chemiluminescence (#20-500, Biological Industries).

### Knocking-Down and Knocking-Out Target Genes

Knock-down (KD) of CXCL8, p65 (RELA) and NOTCH1 by transient siRNA transfections was performed in MDA-MB-231 cells and/or MSCs, as appropriate, using the Lipofectamine RNAiMAX transfection reagent (#56531; Invitrogen, Grand Island, NY) according to manufacturer's instructions. The following ON-TARGET plus siRNA SMART pools were used (all from Dharmacon, Lafayette, CO): Human CXCL8: #L-004756-00; p65: #L-003533-00; NOTCH1: #L-007771-00. siRNA control was introduced by ON-TARGET plus non-targeting control siRNA pool (#D-001810-10). After 24 h, the cells were used in assays, as necessary. Down-regulation of CXCL8 was validated by ELISA or qRT-PCR, as appropriate; p65 and Notch1/N1-ICD down-regulation was validated by WB.

Knock-out (KO) of p65 (RELA) in MDA-MB-231 cells was introduced by lentiviral infection using the clustered regularly-interspaced short palindromic repeats associated protein endonuclease 9 (CRISPR/Cas9) system [as in Danziger et al. (48)]. Two different single guide RNAs targeting p65 were used (sgRNA1: 5′-AGCGCCCCTCGCACTTGTAG-3′; sgRNA2: 5′-CAAGTGCGAGGGGCGCTCCG-3′). Validation of p65 KO in single clones was determined by WB; then, 3 different single cell clones (2 clones expressing sgRNA1 and 1 expressing sgRNA2) were selected to generate a KO-p65-MDA-MB-231 cell pool. In parallel, strand targeting green fluorescent protein (GFP) (5′-GGGCGAGGAGCTGTTCACCG-3′) was used to generate control KO-GFP-MDA-MB-231 cell pool, as above. Efficiency of p65 KO of pooled cells was determined by WB.

### Statistical Analyses

The statistical analyses of the TCGA patient dataset were described in their respective section. *In vitro* experiments were performed in *n* ≥ 3 independent experimental repeats, with MSCs from ≥2 different donors, as indicated in respective figure legends. The results of ELISA, qRT-PCR, WB, migration and invasion assays were compared by two-tailed unpaired Student's *t*-test. Values of *p* ≤ 0.05 were considered statistically significant. Adjustment for multiplicity of comparisons was done using the Benjamini-Hochberg procedure controlling the FDR at 0.05. All the significant results remained statistically significant after correcting for their multiplicity, except for some of the WB results in **Figure 9**. In these latter cases lack of significance was due to high variance between the intensities of effects of the experimental repeats of the test, despite the fact that they all demonstrated the same trend.

## Results

### DAPT Inhibits the High Migratory and Invasive Properties Acquired by TNBC Cells Following Their Interaction With Stromal Cells in the Context of Pro-inflammatory Stimulation

In our previous study, we demonstrated that MDA-MB-231 TNBC cells acquired an increased migratory and invasive potential following their interactions with MSCs and CAFs, in the presence of TNFα (26). To determine if the Notch pathway regulates these processes, TNFα-stimulated MDA-MB-231:MSC and MDA-MB-231:CAF co-cultures were established and migration and/or invasion assays were performed in the presence or absence (control DMSO-treated cells) of DAPT, a potent inhibitor of γ-secretase that participates in the activation of all Notch receptors (49–51).

**Figure 1:**
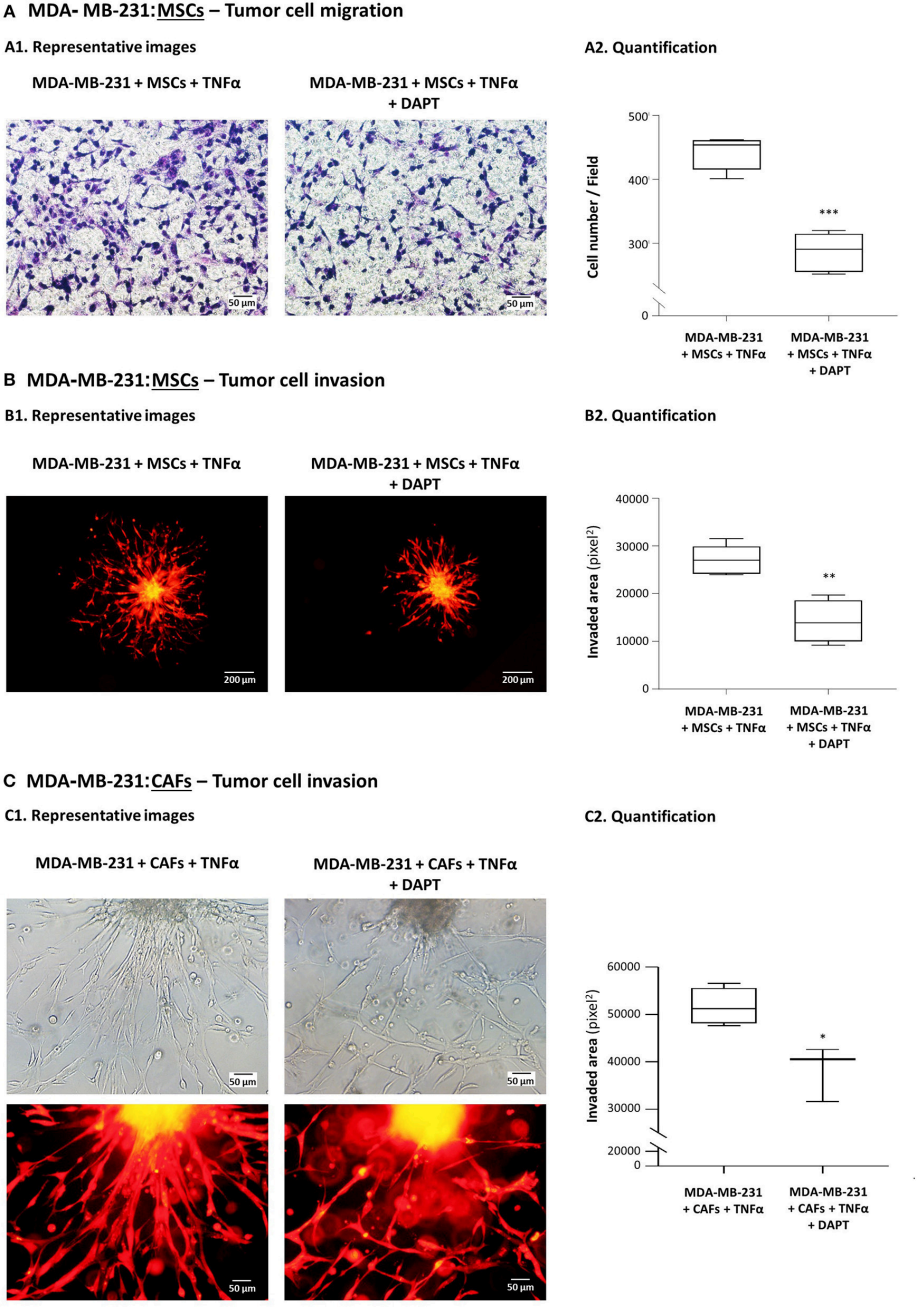
DAPT inhibits the migratory and invasive properties gained by TNBC cells following their interactions with MSCs in the presence of TNFα stimulation. **(A)** Tumor cell migration. mCherry-MDA-MB-231 cells and MSCs were cultured together in migration transwells in the presence of TNFα (10 ng/ml), with DAPT (10 μM) or with its vehicle control (DMSO) in serum-free media. Tumor cell migration was determined toward medium containing 10% FBS, after 12 h. Comparisons of migration of MDA-MB-231 cells following interactions with MSCs and TNFα stimulation to migration of the tumor cells grown in control conditions (without MSCs and TNFα) were presented in our previous study (26). In the current Figure: **(A1)** Representative photos (Bar, 50 μm) and **(A2)** quantifications of multiple photos by ImageJ are provided. ***p < 0.001. The photos and their quantifications are representatives of *n* = 3 independent experiments, performed with MSCs of 2 different donors. Parallel photos taken by fluorescence microscope indicated that migrating cells expressed mCherry, and thus consisted of tumor cells (Supplementary Figure 1). **(B,C)** Tumor cell invasion out of matrigel-embedded 3D spheroids. Spheroids containing mCherry-expressing MDA-MB-231 cells together with MSCs **(B)** or with breast cancer patient-derived CAFs **(C)** were formed in the presence of DAPT (10 μM) or its vehicle (DMSO). Then, spheroids were embedded in matrigel, were stimulated by TNFα (10 ng/ml) and supplemented with fresh DAPT (10 μM) or DMSO. Comparisons of invasion of MDA-MB-231 cells following interactions with MSCs and TNFα stimulation to invasion of the tumor cells grown in control conditions (without MSCs and TNFα) were presented in our previous study (26). In the current Figure: **(B1,C1)** Representative photos (Bar: 200 μm in **B1**, 50 μm in **C1**) and **(B2,C2)** quantifications of multiple photos by ImageJ are provided. **p < 0.01, *p < 0.05. The photos and their quantifications are representatives of *n* > 3 independent experiments, in Part **(B)** performed with MSCs of 2 different donors.

The findings of Figure 1A indicate that the migration of mCherry-MDA-MB-231 cells that interacted with MSCs in the presence of TNFα was markedly inhibited by DAPT (mCherry signals, showing that the migrating cells were tumor cells, are demonstrated in Supplementary Figure 1). Moreover, much of the invasive advantages that were endowed to the tumor cells by their co-culturing with MSCs in the context of TNFα stimulation (26), were inhibited by DAPT (Figure 1B). In parallel, in TNFα-stimulated spheroids of co-cultured MDA-MB-231 cells with breast cancer patient-derived CAFs, reduced ability to invade was revealed upon DAPT treatment (Figure 1C2); in addition, a marked change in the invasion pattern was noted after inhibition of the Notch pathway: The organized and directional motility of control cells (untreated by DAPT) has diverted into a dis-ordered and non-orchestrated phenotype in the presence of DAPT (Figure 1C1).

### DAPT Inhibits the Contact-Dependent Induction of CXCL8, but Not of CCL5 in TNBC:Stroma Co-cultures Stimulated by Pro-inflammatory Cytokines

In our companion study (26) we demonstrated that TNFα and IL-1β stimulation of TNBC:MSC “Contact” co-cultures has led to exacerbated release of CXCL8 and CCL5, more than in non-stimulated “Contact” co-cultures, in cytokine-stimulated/non-stimulated individual cells and in “Transwell” co-cultures (for readers' convenience, Supplementary Figures 2A,B demonstrate the entire panel of cells and stimulations that was provided in our previous study for CXCL8 and CCL5, respectively; different experiments are demonstrated in the two papers). We also found that the induction of CXCL8 was mediated by physical contacts between the two cell types as well as by exchange of soluble factors between them, whereas the induction of CCL5 was entirely contact-dependent [(26); Supplementary Figures 2A,B].

**Figure 2:**
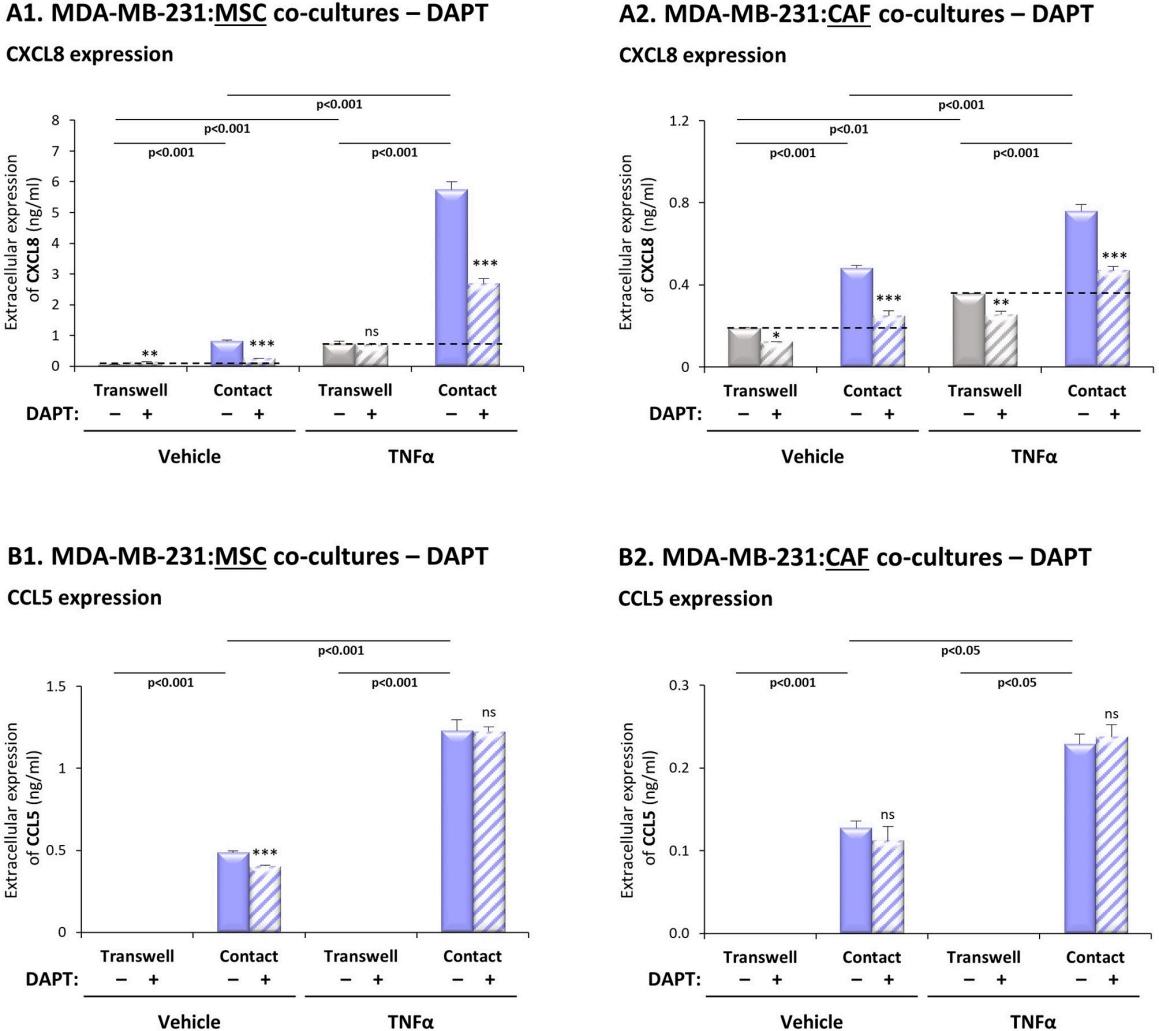
DAPT inhibits the contact-dependent induction of CXCL8, but not of CCL5, in TNFα-stimulated TNBC:stroma co-cultures. Co-cultures of MDA-MB-231 cells with MSCs **(A1,B1)** and co-cultures of MDA-MB-231 cells with breast cancer patient-derived CAFs **(A2,B2)** were established under “Transwell” conditions and “Contact” conditions. Co-cultures were stimulated by TNFα (10 ng/ml) or vehicle control for 7 h; then, TNFα stimulation was removed and the cells were grown in TNFα-free media with DAPT (10 μM) for additional 60 h. CM were collected and the extracellular expression of CXCL8 **(A1,A2)** and CCL5 **(B1,B2)** was determined by ELISA. ***p < 0.001, **p < 0.01, *p < 0.05, ns = non-significant for differences between DAPT- and DMSO-treated cells, within each group. The results are of a representative experiment of *n* > 3 independent experiments, performed with MSCs of 2 different donors, and of *n* = 3 independent experiments performed with patient-derived CAFs. To provide the readers with the entire setup of co-culture conditions compared to separate cells, as we have demonstrated in our previous study (26), Supplementary Figures 2A,B; demonstrate the entire panel of cells/stimulations relevant to CXCL8 and CCL5 induction (without DAPT treatment). The results presented in Supplementary Figure 2 were derived from a different experiment than those presented in our previous study.

To investigate the roles of the Notch pathway in regulating the contact-dependent process of CXCL8 and CCL5 induction in our system, “Contact” and “Transwell” TNBC:MSC co-cultures were stimulated by TNFα in the presence of DAPT or its DMSO control; then, CXCL8 and CCL5 levels in TNFα-free CM were determined (as described in “Materials and methods”). Here, we focused on the ability of DAPT to inhibit the amount of CXCL8 and CCL5 added to “Contact” conditions compared to “Transwell” conditions, as this increment in chemokine release (Figure 2A and Supplementary Figure 2A; for CXCL8: above the dashed lines) was due to physical contacts that were formed between the two cell types.

The results of Figure 2A1 indicate that DAPT caused pronounced inhibition of the contact-dependent increase in CXCL8 (above the dashed line), when MDA-MB-231 cells interacted with MSCs in the presence of TNFα stimulation, and also without TNFα stimulation. To follow up on reports on high heterogeneity of TNBC cells (3), our analyses of two additional TNBC cell lines (Supplementary Table 2) demonstrated less pronounced effects of DAPT on TNFα-stimulated MDA-MB-468:MSC “Contact” co-cultures, and no consistent effects of DAPT on the responses of BT-549:MSC “Contact” co-cultures stimulated by TNFα.

Additional experiments have further supported the roles of the Notch pathway in up-regulating CXCL8 expression by TNBC-stroma-inflammation networks: First, similar to MDA-MB-231:MSC co-cultures stimulated by TNFα (Figure 2A1), DAPT has inhibited CXCL8 elevations in TNFα-stimulated co-cultures of MDA-MB-231 cells with patient-derived CAFs (Figure 2A2). Second, analyses that were performed on IL-1β-stimulated co-cultures of TNBC cells (MDA-MB-231, MDA-MB-468, BT-549) with MSCs and/or CAFs (Supplementary Figure 3A; Supplementary Table 2) demonstrated generally a similar pattern to the findings obtained by TNFα stimulation.

In contrast to this mode of regulation in CXCL8, the contact-dependent process of CCL5 induction in TNFα-stimulated MDA-MB-231:MSC and in MDA-MB-231:CAF co-cultures, was not affected by DAPT treatment (Figure 2B). Of note, parallel experiments that were performed with IL-1β-stimulated MDA-MB-231:MSC and MDA-MB-231:CAF co-cultures have shown similar findings to those with TNFα (Supplementary Figure 3B).

### In TNFα-Stimulated TNBC:MSC Co-cultures, Mainly TNBC Cells but Also MSCs, Contribute to Elevations in CXCL8 Expression, Through a p65-Depenent Process

Following the above observations, we set to determine the molecular mechanisms regulating CXCL8 expression in the tumor-stroma-inflammation network, and to reveal the roles and regulation of Notch receptors in this setting. We began this part of the study by asking which of the two cell types, the MSCs and/or the tumor cells, contribute/s to CXCL8 expression in TNFα-stimulated MDA-MB-231:MSC “Contact” co-cultures. In addition, in view of our previous observations on p65 activation by TNFα and IL-1β in both cell types [(26); Supplementary Figure 2C] we asked if p65 controls CXCL8 transcription, and in which of the two cell types this regulation takes place.

**Figure 3:**
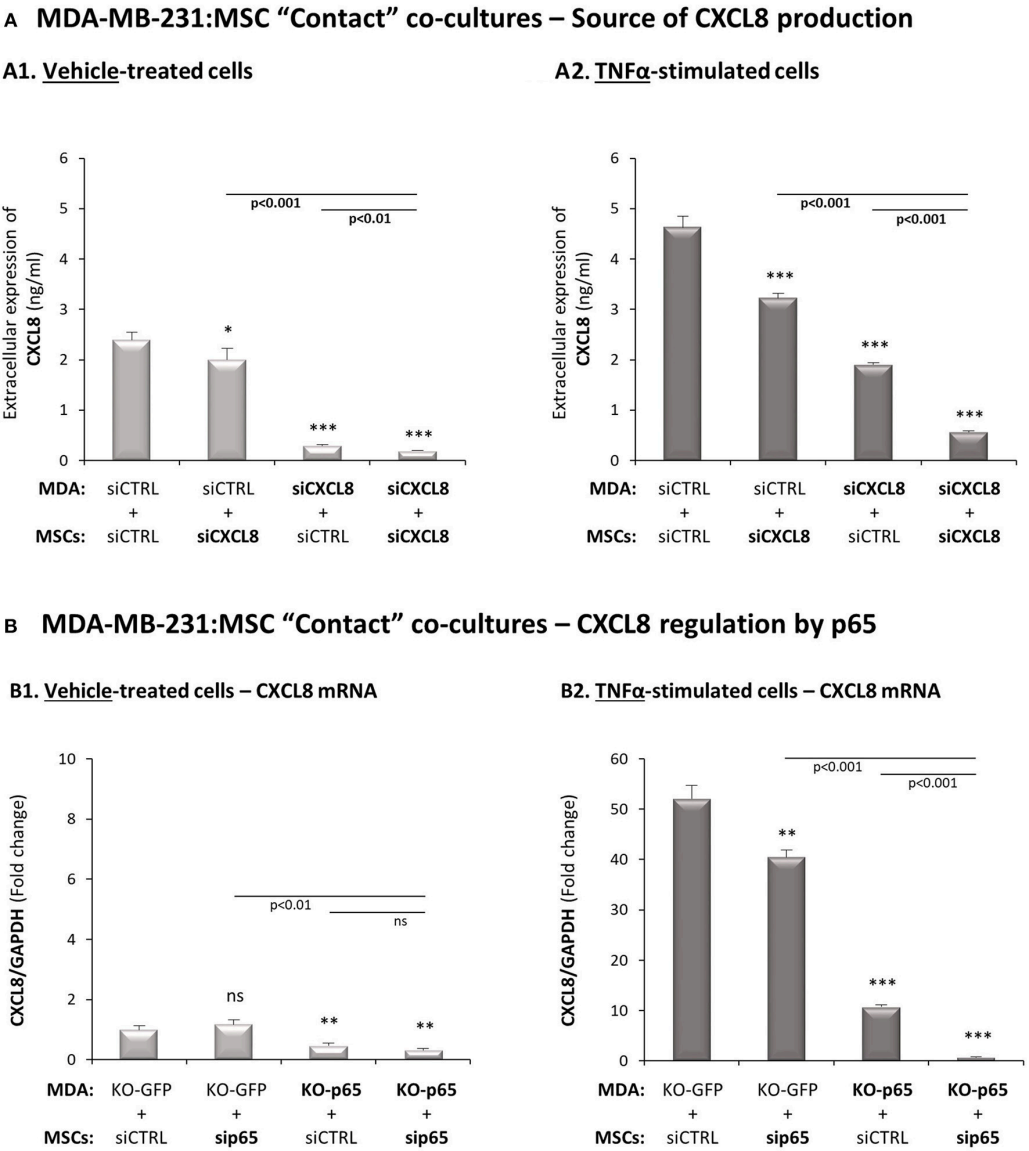
In TNFα-stimulated TNBC:MSC “Contact” co-cultures, mainly TNBC cells but also MSCs contribute to elevated levels of CXCL8, through a p65-dependent process. **(A)** The cellular source of CXCL8. siRNA to CXCL8 was expressed in MDA-MB-231 cells (“MDA”), in MSCs or both cell types together (validation of CXCL8 down-regulation is demonstrated in Supplementary Figure 4A), and “Contact” co-cultures were established in the presence of vehicle **(A1)** or TNFα (10 ng/ml) **(A2)**. CXCL8 levels in cell supernatants were determined by ELISA, as described in Figure 2. ***p < 0.001, *p < 0.05 for differences between co-culture combinations that included siCXCL8-expressing cells, compared to co-cultures in which both cell types expressed siCTRL. The results are of a representative experiment of *n* = 3 independent experiments, performed with MSCs of 2 different donors. **(B)** Regulation of CXCL8 expression by p65. p65 was down-regulated in MDA-MB-231 cells (“MDA”), MSCs or both cell types together (validations of p65 down-regulation are demonstrated in Supplementary Figures 4B,C), and “Contact” co-cultures were established and were exposed to vehicle **(B1)** or to TNFα (10 ng/ml) **(B2)**. CXCL8 mRNA levels were determined by qRT-PCR. ***p < 0.001, **p < 0.01, ns=non-significant for differences between different sip65/siCTRL and KO-GFP/KO-p65 cell combinations compared to siCTRL/KO-GFP control groups. The results are of representative experiment of *n* > 3 independent experiments, performed with MSCs of 3 different donors.

To determine the cellular source of CXCL8, the expression of the chemokine was knocked-down by siRNA in MDA-MB-231 cells, in MSCs or in both cell types together during the co-culture process, in “Contact” conditions. Then, we determined the levels of CXCL8 produced with and without TNFα stimulation (Supplementary Figure 4A demonstrates high efficacy of CXCL8 down-regulation in both cell types). When MDA-MB-231:MSC co-cultures were established without TNFα stimulation, the tumor cells were almost the exclusive source for the chemokine (Figure 3A1). However, following stimulation by TNFα, the equilibrium between the two cell types was changed: not only the tumor cells but also MSCs - although at lower levels - contributed to the elevation in CXCL8 expression by the cytokine-stimulated co-cultures (Figure 3A2). Similar findings were noted following IL-1β-induced TNBC:MSC stimulation, as demonstrated in Supplementary Figure 5.

Next, p65 was knocked-down in the MSCs by siRNA and was knocked-out in the tumor cells by CRISPR/Cas9, leading to efficient reduction in p65 expression and activation in both cell types (Supplementary Figures 4B,C; although p65 activation was not down-regulated completely in MSCs, it was sufficient to clearly reveal the mechanistic roles of p65 in the studied processes, as shown below). The data of Figure 3B indicate that in MDA-MB-231:MSC “Contact” co-cultures that had not been stimulated by TNFα, p65 basal activation in the tumor cells was the sole inducer of CXCL8 expression. However, in TNFα-stimulated co-cultures, p65-regulated CXCL8 expression was partially contributed by MSCs, even though most of the p65-induced CXCL8 expression was contributed by the tumor cells (Figure 3B2). Most importantly, when p65 was down-regulated in both cell types together, almost no CXCL8 was produced by the “Contact” co-cultures indicating that p65 was the master regulator of CXCL8 expression in this setting of the tumor-stroma-inflammation network.

### Analyses of Notch Receptors Point at Notch1 as Possible Regulator of CXCL8 Induction in the Tumor-Stroma-Inflammation Networks in TNBC

**Figure 4:**
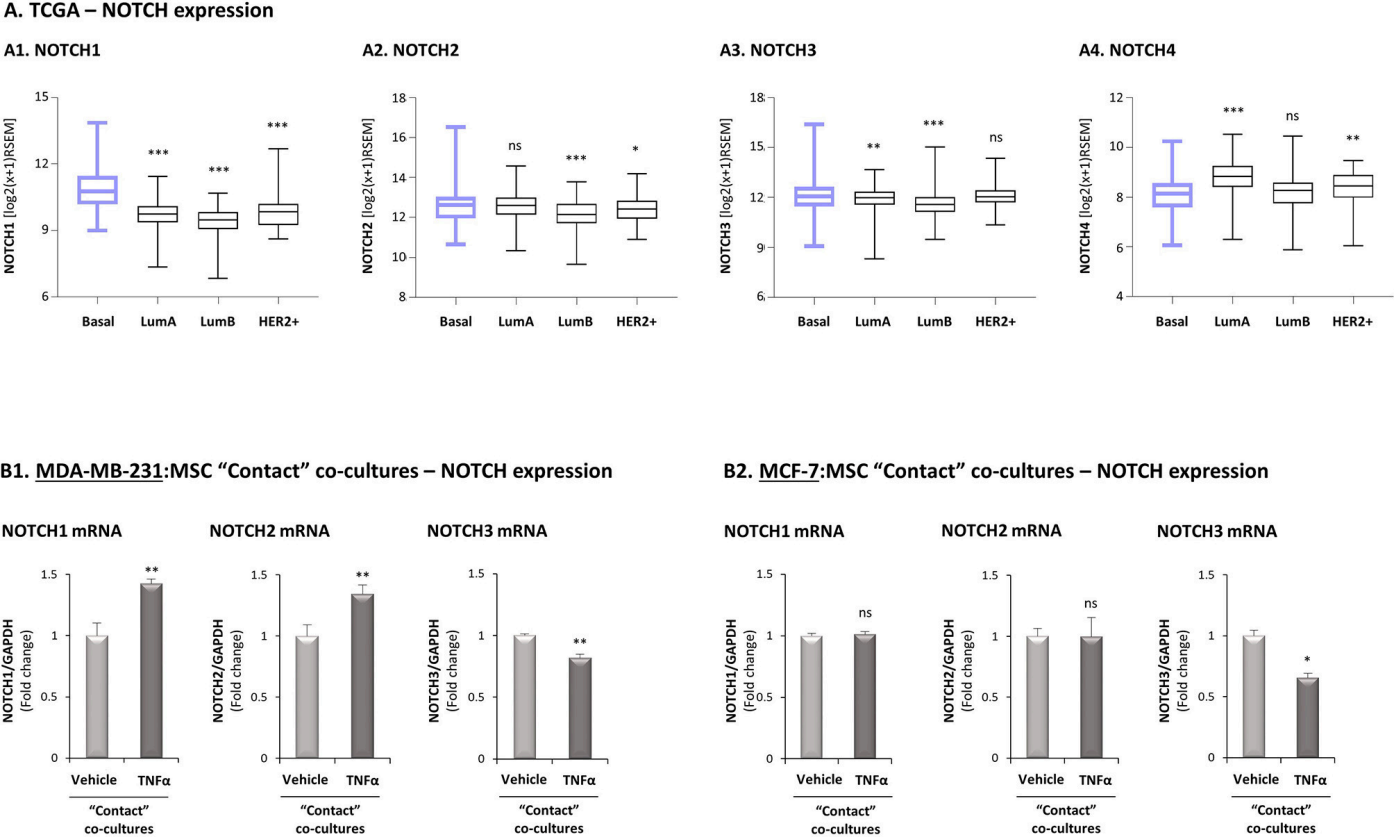
Studies of the TCGA breast cancer patient dataset and of transcriptional regulation demonstrate high relevance of Notch1 to the tumor-stroma-inflammation network in TNBC. **(A)** The Figure demonstrates gene expression boxplot analyses, performed using the TCGA breast cancer patient dataset. **(A1)** NOTCH1. **(A2)** NOTCH2. **(A3)** NOTCH3. **(A4)** NOTCH4. ***p < 0.001, **p < 0.01 *p < 0.05, ns = non-significant for differences between the expression of NOTCH receptors in luminal-A, luminal-B and HER2+ tumors compared to basal tumors. **(B)** mRNA expression levels of NOTCH1, NOTCH2 and NOTCH3 were determined by qRT-PCR in MDA-MB-231:MSC **(B1)** or MCF-7:MSC **(B2)** “Contact” co-cultures stimulated by TNFα (10 ng/ml) or vehicle control. **p < 0.01, *p < 0.05, ns=non-significant for differences between cytokine-stimulated and non-stimulated co-cultures. The results are of a representative experiment of *n* ≥ 3 independent experiments, performed with MSCs of 2 different donors.

To provide additional molecular insights into processes that possibly connect Notch receptors to CXCL8 induction in the TNBC-stroma-inflammation network, we next assessed the potential relevance of each of the four human Notch receptors to the research systems of our study. First, in view of our previous findings demonstrating that the tumor-stroma-inflammation network is more effective in TNBC than in the less aggressive subtype of breast cancer, luminal-A (26), we asked if the expression of any specific Notch receptor is significantly elevated in TNBC tumors compared to the luminal-A tumors. We also compared the expression of Notch receptors in basal tumors to luminal-B and HER2+ tumors, in order to identify Notch receptors whose elevated expression signifies more clearly the basal subtype of breast cancer. Using the TCGA breast cancer dataset, we found that the expression of NOTCH1 was significantly higher in basal tumors than in all other subtypes of disease (Figure 4A1). In contrast, NOTCH2 expression levels were similar in basal and luminal-A tumors (Figure 4A2), and NOTCH3 was similarly expressed in basal tumors and HER2+ tumors (Figure 4A3). Of note, NOTCH4 expression was lower in basal tumors than in tumors of the less aggressive luminal-A subtype and of the HER2+ subtype (Figure 4A4).

**Figure 5:**
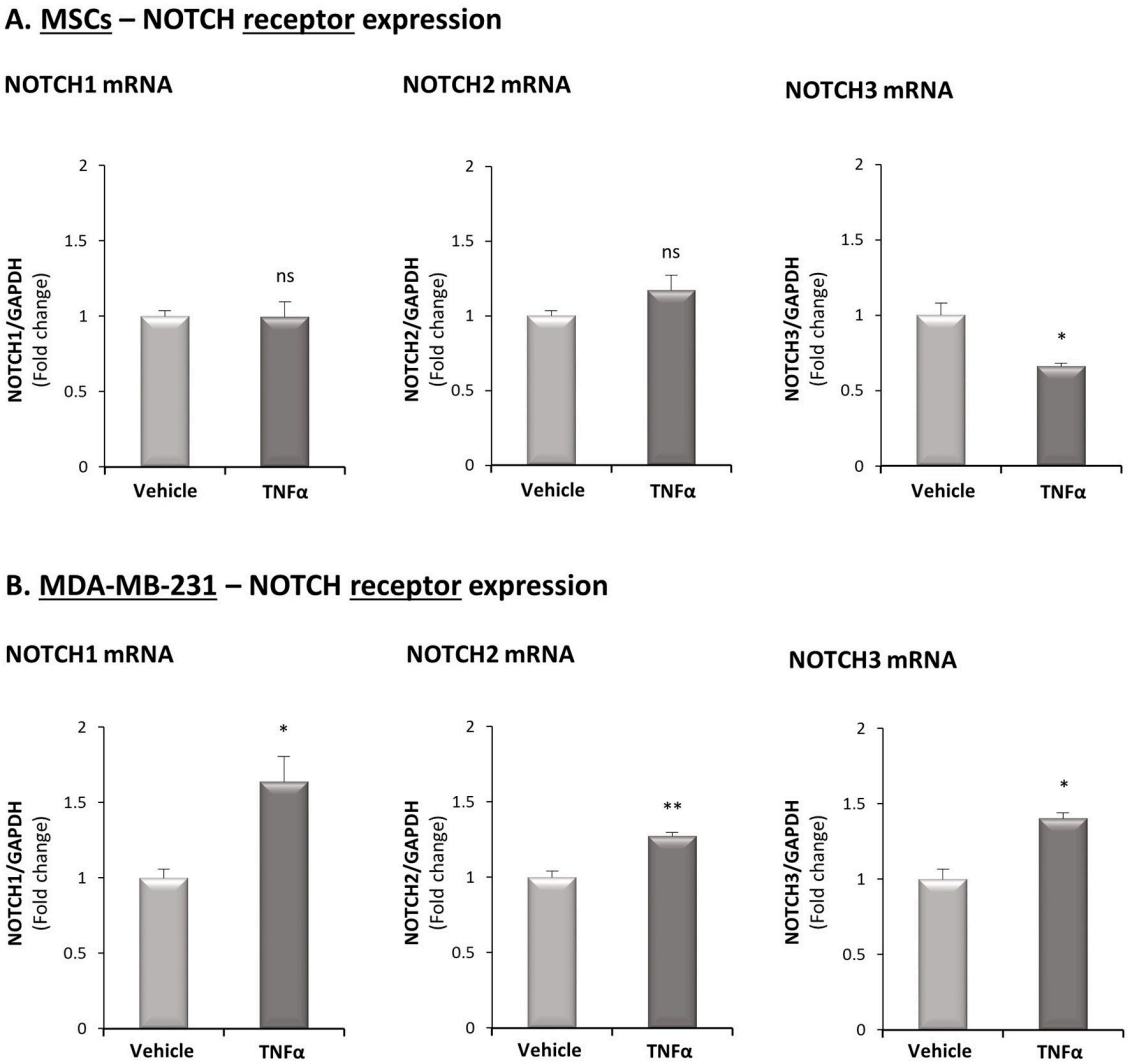
TNFα up-regulates the expression of NOTCH1, NOTCH2 and NOTCH3 in TNBC cells but not in MSCs. The Figure demonstrates mRNA expression of NOTCH1, NOTCH2 and NOTCH3 in MSCs **(A)** and in MDA-MB-231 cells **(B)**, following stimulation by TNFα (10 ng/ml) or vehicle control. **p < 0.01, *p < 0.05, ns = non-significant for differences between cytokine-stimulated and vehicle-treated cells. The results are of a representative experiment of *n* ≥ 3 independent experiments, performed with MSCs of 2 different donors.

We next determined in TNFα-stimulated MDA-MB-231:MSC “Contact” co-cultures the levels of Notch receptors. Here, we found that NOTCH1 and NOTCH2 mRNA levels were significantly elevated by TNFα stimulation, whereas the levels of NOTCH3 were somewhat reduced (Figure 4B1; Based on the TCGA results, NOTCH4 was not analyzed). Unlike these findings on NOTCH1 and NOTCH2 regulation by TNFα in TNBC:MSC co-cultures, TNFα stimulation of luminal-A MCF-7:MSC “Contact” co-cultures did not lead to NOTCH1 and NOTCH2 elevations (Figure 4B2). Cell-specific analyses complemented these results by indicating that NOTCH1, NOTCH2 and NOTCH3 were up-regulated by TNFα in the tumor cells but not in the MSCs (Figure 5). Of note, the findings described above - mainly those on NOTCH1 up-regulation following TNFα stimulation of MDA-MB-231:MSC “Contact” co-cultures and of each cell type alone - were in general similarly produced by IL-1β stimulation (Supplementary Figure S6).

**Figure 6:**
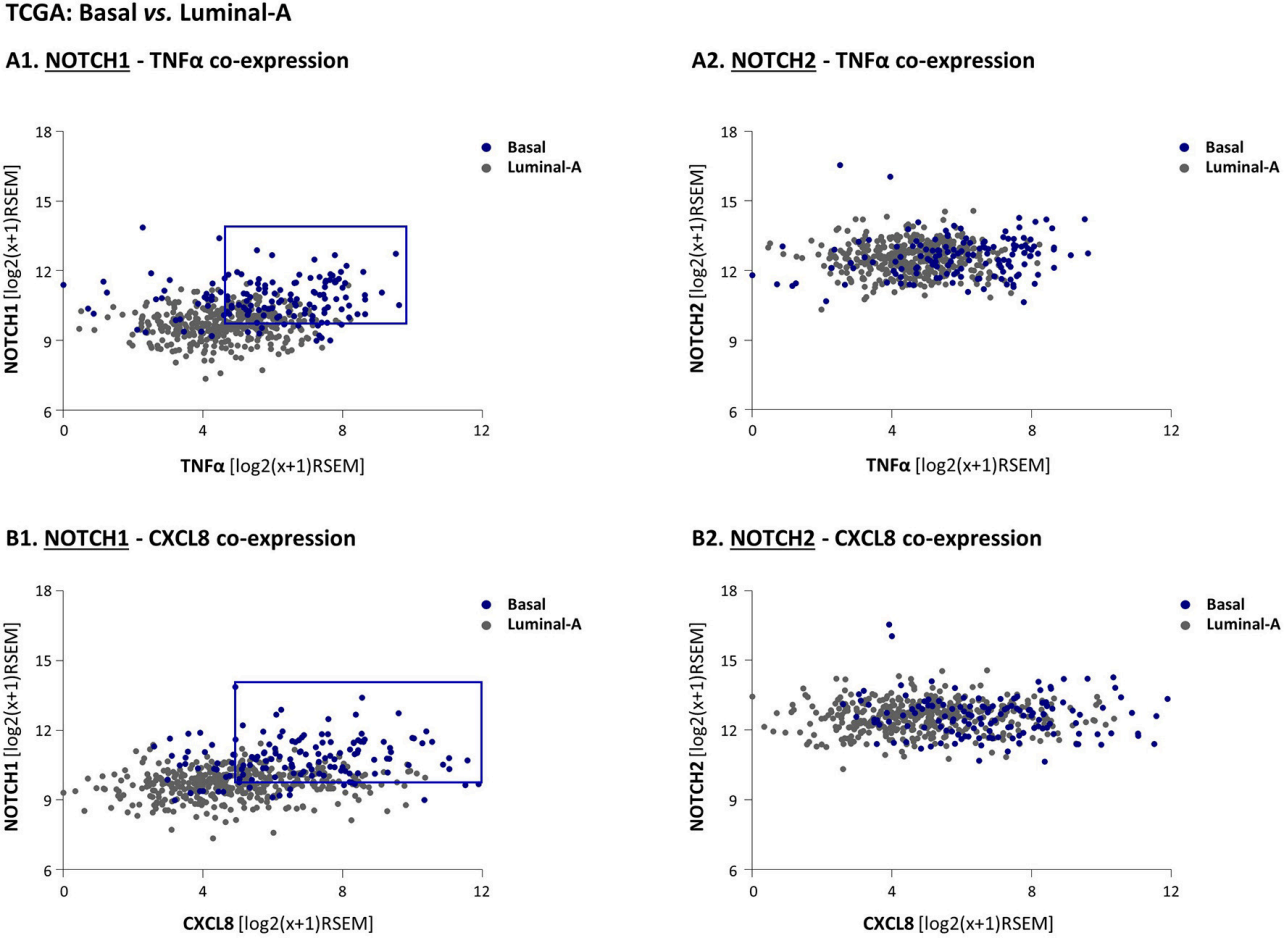
High co-expression levels of NOTCH1 - but not of NOTCH2 - with TNFα and CXCL8 differentiate basal patients from luminal-A breast cancer patients. **(A,B)** The Figure demonstrates gene expression analyses, performed using the TCGA breast cancer dataset. The analyses demonstrate in individual patient tumor (dots) the co-expression of NOTCH1 with TNFα **(A1)**; NOTCH2 with TNFα **(A2)**; NOTCH1 with CXCL8 **(B1)**; NOTCH2 with CXCL8 **(B2)**. The blue rectangle illustrates the upwards-right shift observed in basal patients compared to luminal-A patients in NOTCH1 co-expression analyses (the rectangle was set as described in “Materials and methods”).

To follow up on these findings, suggesting that Notch1, and possibly also Notch2 may be connected to the cytokine-stimulated integrative system that we study in TNBC, we next used the TCGA dataset to analyze the relevance of Notch1 and Notch2 to the tumor-stroma-inflammation network. To this end, we determined the co-expression patterns of NOTCH1/NOTCH2 with two major players in this setting: TNFα and its target, CXCL8. Specifically, we asked which of the Notch receptor-related co-expression patterns would dissociate the basal subtype from the luminal-A subtype. To this end, the expression levels of NOTCH1 and TNFα in each individual patient tumor were plotted, in both groups of patients. The findings of Figure 6A1 indicate that in general, basal tumors co-expressed higher levels of NOTCH1 and TNFα than luminal-A tumors as demonstrated by an upward-right shift compared to luminal-A patients (marked by blue rectangle, set as described in “Materials and methods”), thus dissociating the basal patients from the luminal-A patients. In contrast, the pattern of NOTCH2-TNFα co-expression in basal patients overlapped with the co-expression pattern in luminal-A patients, and thus did not dissociate between these two groups of patients (Figure 6A2). As with TNFα, the co-expression pattern of NOTCH1 but not of NOTCH2, with CXCL8, has differentiated the basal patients from the luminal-A patients (Figure 6B). The higher relevance of Notch1 than of Notch2 to the tumor-stroma-inflammation networks in TNBC was corroborated by similar findings that were obtained with NOTCH1 *vs*. NOTCH2 co-expression analyses performed with IL-1β (Supplementary Figure 7).

### Notch1 Activation Is Required for the Contact-Dependent Induction of CXCL8 in the Tumor-Stroma-Inflammation Network in TNBC

**Figure 7:**
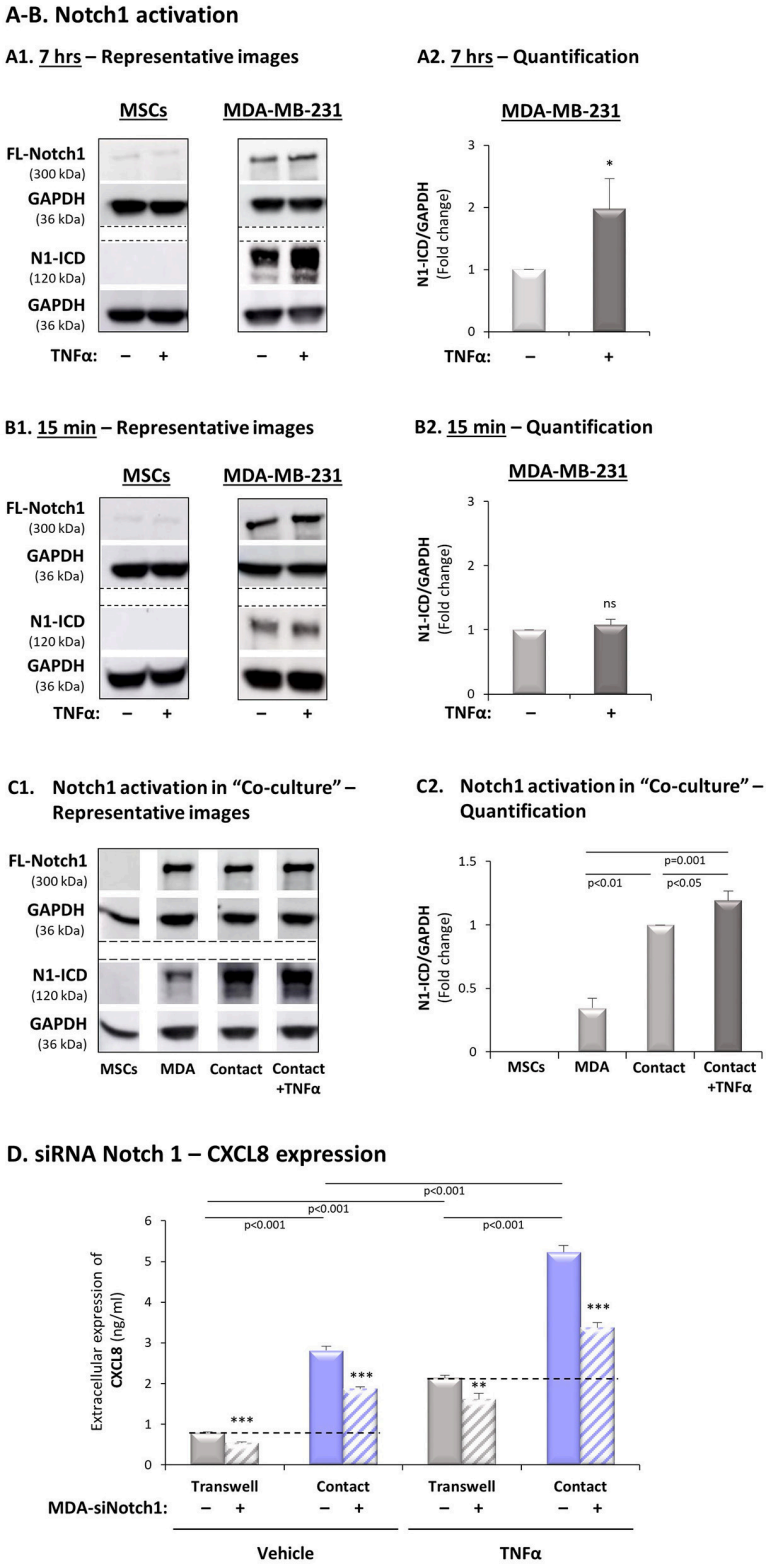
Expression of Notch1 siRNA in TNBC cells inhibits the contact-dependent induction of CXCL8, in TNFα-stimulated TNBC:MSC co-cultures. **(A,B)** Notch1 activation by TNFα stimulation in MSCs and MDA-MB-231 cells. Each of the two cell types was stimulated by TNFα (“+”; 10 ng/ml) or vehicle control (“–“) for 7 h **(A)** or 15 min **(B)**. **(A1,B1)** Representative experiments and **(A2,B2)** averages ± SD values of Notch1 activation in *n* ≥ 3 independent experiments, performed with MSCs of 2 different donors. Notch1 activation levels were determined by WB analyses of N1-ICD = Notch1 intracellular domain. FL-Notch1 = Full-length Notch1. GAPDH was used as a loading control. *p < 0.05, ns = non-significant for differences between cytokine-stimulated cells and control non-stimulated MDA-MB-231 cells (MSCs are not presented in the graphs because no N1-ICD signals were detected). **(C)** Notch1 activation was determined in MDA-MB-231 (“MDA”) cells, MSCs and “Contact” co-cultures that were stimulated by TNFα (10 ng/ml), or exposed to vehicle for 7 h. **(C1)** A representative experiment and **(C2)** averages ± SD of Notch1 activation in *n* = 3 independent experiments, performed with MSCs of 3 different donors. WB analyses were performed as described in Part **(A)** above. **(D)** The effects of siRNA Notch1 on CXCL8 expression (validation of Notch1 down-regulation is demonstrated in Supplementary Figure 4D). MDA-MB-231 cells (“MDA”) transfected by Notch1 siRNA (“+”) or siCTRL (“–”) and were co-cultured with MSCs. Then, co-cultures were stimulated by TNFα (10 ng/ml) or by vehicle control, and extracellular expression of CXCL8 was determined by ELISA, as described in Figure 2. ***p < 0.001, **p < 0.01 for differences between groups containing siNotch1-expressing MDA-MB-231 cells and groups of siCTRL-expressing MDA-MB-231 cells. The results are of a representative experiment of *n* = 3 independent experiments, performed with MSCs of 2 different donors.

Based on the above findings, pointing at Notch1 as a potential regulator of the TNBC-stroma-inflammation networks, we determined the roles of Notch1 in regulating the contact-dependent process of CXCL8 induction in TNFα-stimulated MDA-MB-231:MSC co-cultures. First, we asked if MDA-MB-231 cells and/or MSCs respond to TNFα stimulation by Notch1 activation. Using Abs that specifically recognize the N1-ICD, the cleaved and activated form of Notch1 (directed to Val1744, which is exposed in Notch1 following γ-secretase-mediated cleavage), we found that Notch1 underwent basal process of activation in MDA-MB-231 cells (Figures 7A,B). Then, we noticed that the N1-ICD levels in MDA-MB-231 cells were significantly elevated upon 7 h but not after 15 min of TNFα stimulation (Figures 7A,B, respectively). Thus, a time-dependent TNFα-induced process of Notch1 activation was revealed in MDA-MB-231 cells. In contrast, no evidence for basal Notch1 activation or for its induction by TNFα stimulation was observed in MSCs at any of the time points (Figures 7A,B). Similar results were observed following IL-1β stimulation, demonstrating that 7-h stimulation, but not 15-min stimulation by IL-1β has led to Notch1 activation in the tumor cells, but not in the MSCs (Supplementary Figures 8A,B). Furthermore, we noted that the levels of activated N1-ICD were much increased in MDA-MB-231:MSC “Contact” co-cultures compared to those of the tumor cells or MSCs when grown individually; the high N1-ICD levels in “Contact” co-cultures were slightly elevated by TNFα stimulation (Figure 7C).

To follow up on the findings described above we asked if Notch1 knock-down in MDA-MB-231 cells by siRNA would reduce the contact-dependent induction of CXCL8 in TNFα-stimulated MDA-MB-231:MSC co-cultures. To this end, we have analyzed the expression of CXCL8 in TNFα-stimulated and non-stimulated MDA-MB-231:MSC co-cultures in which Notch1 was down-regulated by siRNA in the tumor cells. After validating that Notch1 expression and activation was significantly down-regulated by the siRNA (Supplementary Figure 4D), we found that the contact-dependent induction of CXCL8 upon TNFα stimulation - noted by CXCL8 incremental expression in “Contact” *vs*. “Transwell” conditions - was markedly reduced by siRNA to Notch1 (Figure 7D). Although some levels of Notch1 expression remained after its knock-down by siRNA (Supplementary Figure 4D), the degree of Notch1 down-regulation in this setting gave rise to CXCL8 inhibition levels which were similar to those obtained by DAPT, following TNFα stimulation of “Contact” co-cultures (siRNA Notch1: 73 ± 21% in all experimental repeats; DAPT: 70 ± 13% as in Figure 2A1). Moreover, induction of CXCL8 under “Contact” conditions, in the absence of TNFα stimulation, was also partly dependent on Notch1 activation, as in DAPT studies. Of importance, similar findings on Notch1-regulated, contact-dependent CXCL8 induction were also observed following IL-1β stimulation when siRNA to Notch1 was used (75 ± 25%; Supplementary Figure 8C), in similar levels to those obtained upon treatment by DAPT (65 ± 23%; Supplementary Figure 3A1).

**Figure 8:**
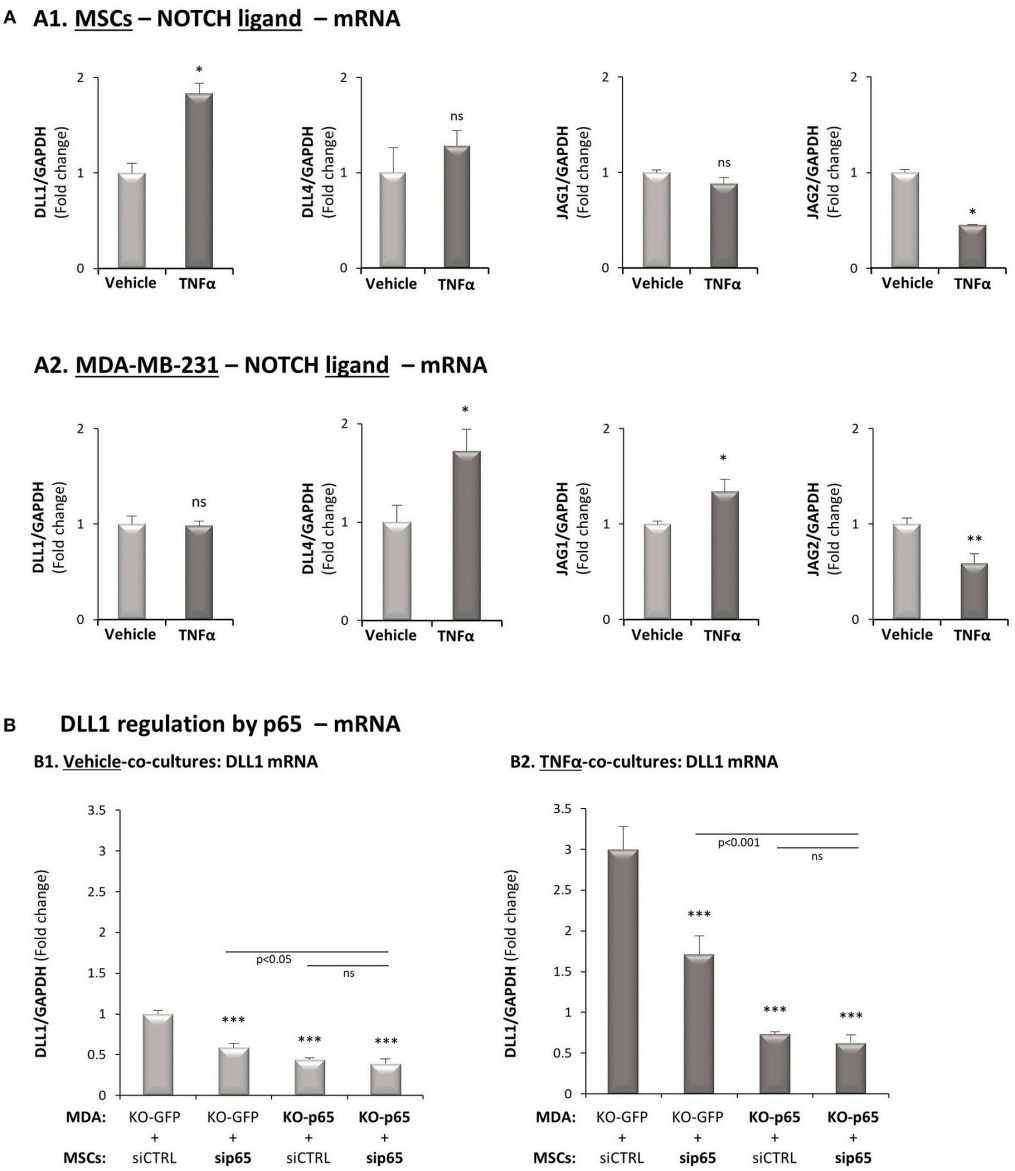
DLL1 is up-regulated in MSCs following TNFα stimulation and is controlled by p65 activation. **(A)** The Figure demonstrates mRNA expression levels of Notch ligands, determined by qRT-PCR in MSCs **(A1)** or in MDA-MB-231 cells **(A2)**, stimulated by TNFα (10 ng/ml) or vehicle control for 7 h. **p < 0.01, *p < 0.05, ns = non-significant for differences between cytokine-stimulated and vehicle-treated co-cultures. **(B)** DLL1 regulation by p65. p65 was down-regulated in MDA-MB-231 cells (“MDA”), MSCs or both cell types together (as in Figure 3B) and both cell types were co-cultured in “Contact” conditions. Then, co-cultures were stimulated by TNFα (10 mg/ml) or exposed to vehicle control, and DLL1 mRNA was determined by qRT-PCR. ***p < 0.001, ns = non-significant. In all parts of the Figure, the results are of a representative experiment of *n* ≥ 3 independent experiments, performed with MSCs of 2 different donors.

To complement the above analyses, clearly indicating that Notch1 activation takes place only in the tumor cells and is required for CXCL8 induction in the TNBC-stroma-inflammation setting, we asked which of the Notch ligands may be a candidate partner that is expressed by MSCs. The analyses presented in Figure 8 indicated that of the different Notch ligands, Delta-like 1 (DLL1) was the only ligand that was up-regulated by TNFα (Figure 8A1); DLL1 expression was also elevated by IL-1β stimulation in the MSCs (Supplementary Figure 6A). It was also interesting to note that following TNFα stimulation, p65 activation was involved in DLL1 up-regulation when MSCs interacted with MDA-MB-231 “Contact” co-culture conditions (Figure 8B). Of note, parallel analyses demonstrated that DLL1 was also up-regulated by p65 activation in MDA-MB-231 cells that interacted with MSCs in the presence of TNFα (Figure 8B2), despite the fact that DLL1 was not up-regulated by TNFα in MDA-MB-231 at all (Figure 8A2).

### In TNFα-Stimulated TNBC:MSC Co-cultures, p65 Activation Mainly in TNBC Cells but Also in MSCs, Induces Notch1 Activation

Our above findings indicated that p65, as well as Notch1, were involved in CXCL8 up-regulation in the TNBC-stroma-inflammation network (Figures 3B, 7D, respectively). Moreover, we demonstrated that p65 was quickly activated by TNFα [15 min; Supplementary Figure 2C and (26)] but TNFα-induced Notch1 activation was slower (close to 7 h; Figures 7A,B). Thus, we determined the possibility that in TNFα-stimulated MDA-MB-231:MSC “Contact” co-cultures, p65 activation had up-regulated Notch1 expression and/or Notch1 activation.

**Figure 9:**
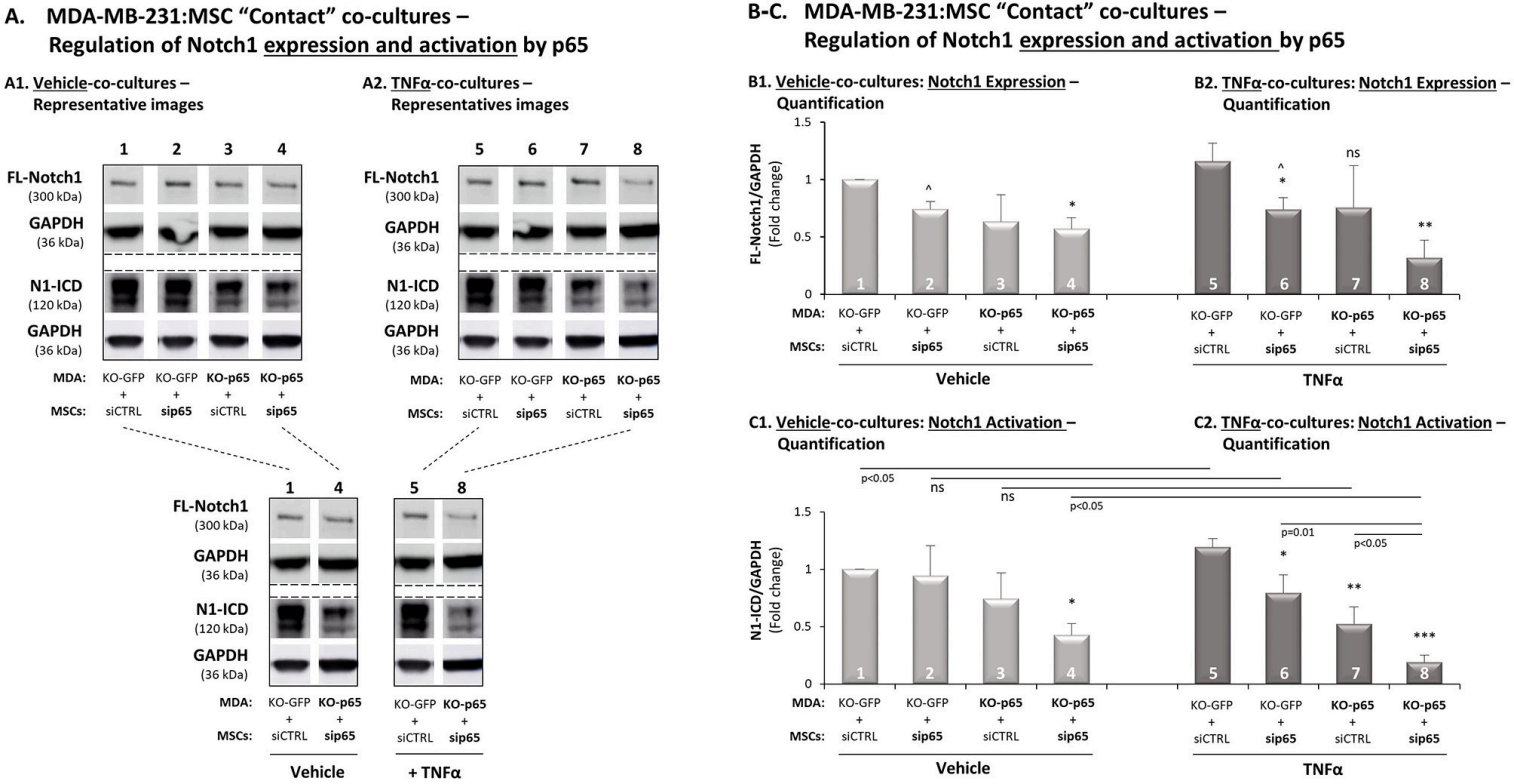
In TNFα-stimulated TNBC:MSC co-cultures, p65 activation up-regulates Notch1 expression and activation. The Figure demonstrates studies performed following p65 down-regulation in MDA-MB-231 cells (“MDA”), MSCs or both cell types together, as described in Figure 3B (validations of p65 down-regulation are demonstrated in Figures S4B,C). **(A)** A representative experiment, **(B)** averages ± SD of Notch1 expression and **(C)** averages ± SD of Notch1 activation in *n* = 3 independent experiments (∧ In these groups, densitometry was performed in *n* = 2), performed with MSCs of 3 different donors. The two cell types were co-cultured in “Contact” conditions in different combinations, and were exposed to vehicle control **(A1,B1,C1)** or to TNFα stimulation (**A2,B2,C2**; 10 ng/ml). Notch1 activation levels were determined by WB analyses of N1-ICD = Notch1 intracellular domain. FL-Notch1 = Full-length Notch1. GAPDH was used as a loading control. ***p < 0.001, **p < 0.01, *p < 0.01, ns = non-significant for differences between different sip65/siCTRL and KO-GFP/KO-p65 cell combinations compared to siCTRL/KO-GFP control groups. For readers' convenience, Panel A also provides another view of the comparisons between specific lanes that were shown in **(A1,A2)**. This approach was taken in order to clearly demonstrate the stronger reduction in Notch1 activation in TNFα-stimulated co-cultures following down-regulation of p65 in both cell types, compared to similar analyses performed without TNFα stimulation or without p65 down-regulation.

By down-regulating p65 in the tumor cells and/or in the MSCs, we demonstrated that in the absence of p65 activation in both cell types together, the expression of Notch1 (FL-Notch1) was significantly reduced, in the presence of TNFα stimulation (Figures 9A,B1; lanes 5 *vs*. 8) and in its absence (Figures 9A,B1; lanes 1 *vs*. 4). Most importantly, based on the N1-ICD bands remaining, we found that the activation of Notch1 was almost completely abrogated following p65 down-regulation in both cell types together when TNBC:MSC interactions took place in the context of TNFα stimulation (Figures 9A,C2; lanes 8 *vs*. 5). In comparison, some degree of Notch1 activation remained upon p65 down-regulation in both cell types together, when similar co-cultures where formed in the absence of TNFα (Figures 9A,C1; lanes 4 *vs*. 1).

Detailed analysis has demonstrated that in the setting that lacked TNFα stimulation, p65 activation mainly in the tumor cells (Figures 9A1,C1; lanes 3 *vs*. 1) but also to some extent in MSCs (Figures 9A1,C1; lanes 2 *vs*. 1) has contributed to Notch1 activation in co-cultures [please note that the differences in this case (lanes 3 *vs*. 1, and lanes 2 *vs*. 1) were detected in all experiments but did not come out statistically significant because of differences in the extent of reduction noted in each of the experimental repeats]. Upon TNFα stimulation of MDA-MB-231:MSC “Contact” co-cultures, it became evident that down-regulation of p65 in the tumor cells has led to marked inhibition of Notch1 activation (Figures 9A2,C2; lanes 7 *vs*. 5), and siRNA to p65 in the MSCs also reduced Notch1 activation, although to much lower extent than in the tumor cells (Figures 9A2,C2; lanes 6 *vs*. 5). Together, these findings indicate that p65 is a most important inducer of Notch1 activation in MDA-MB-231:MSC co-cultures, primarily in the presence of TNFα stimulation.

## Discussion

TNBC tumors are characterized by high aggressiveness, necessitating improved understanding of the mechanisms that promote their progression. In a recent study we identified a tumor-stroma-inflammation network potentiating multiple processes that contribute to increased metastasis in TNBC; they included expression of pro-metastatic chemokines such as CXCL8 and CCL5, angiogenesis and tumor cell migration and invasion. Eventually, this interactive network was shown to give rise to increased metastasis of TNBC cells *in vivo* (26).

Our findings further indicated that cell-to-cell contacts played key roles in potentiating pro-metastatic activities in the tumor-stroma-inflammation setting, leading us to ask if the Notch pathway is involved in promoting pro-metastatic functions when TNBC cells interacted with MSCs in the presence of pro-inflammatory stimulation. Indeed, the findings presented in the current study indicate that the elevated levels of migration and invasion of TNBC cells following their interactions with MSCs/CAFs in the presence of TNFα were mediated by Notch signaling, as these tumor cell functions were prominently inhibited by DAPT. In parallel, we demonstrated that the contact-dependent induction of CXCL8 in cytokine-stimulated “Contact” co-cultures was inhibited by DAPT. Although CCL5 elevation in cytokine-stimulated TNBC:MSC co-cultures was entirely based on cell-to-cell contacts that were established between the two cell types (26), this effect was not inhibited by DAPT. Thus, our findings provide evidence to a novel, Notch-dependent mechanism, which regulates CXCL8 in TNBC, and indicate that this Notch-mediated regulatory mechanism is not shared by all pro-metastatic chemokines, like CCL5.

Moreover, the findings obtained with DAPT were recapitulated when Notch1 was knocked-down in the tumor cells by siRNA; in these experiments, Notch1 siRNA has led to similar reduction in TNFα-induced contact-dependent induction of CXCL8 as did DAPT. Moreover, Notch1 was up-regulated in the tumor cells at the mRNA and protein levels and was activated at the protein level in the tumor cells by TNFα stimulation. These findings and similar results obtained by IL-1β stimulation indicated that Notch1 is the actual Notch receptor involved in the up-regulation of CXCL8, and is activated only in the tumor cells upon TNFα/IL-1β stimulation of TNBC:MSC co-cultures. In further studies it will be interesting to identify the ligands that bind Notch1 in the tumor cells. Based on our findings at the mRNA levels, it is possible that DLL1, whose expression was elevated in MSCs by TNFα and IL-1β stimulation (through a p65-mediated pathway, as analyzed for TNFα stimulation), is a partner of tumor cell-expressed Notch1. However, our findings demonstrating that DLL1 is regulated by p65 also in the cancer cells suggests that reciprocal interactions take place between the two cell types, in a complex manner that requires further investigation.

In line with our observations, published studies indicate that of the four Notch receptors (Notch1-4), Notch1 is strongly linked to disease progression in TNBC. Notch1 was found to be over-expressed and hyper-activated in TNBC patients, and high Notch1 levels were associated with reduced overall survival in TNBC/basal breast cancer patients (45, 52–54). Moreover, meta-analysis of breast cancer studies revealed a significant association between high Notch1 expression and TNBC progression (55). Studies in breast cancer, particularly in the TNBC subtype, demonstrated that the activation of Notch1 promoted stemness, drug resistance, invasion and migration (41–45, 52–58).

Moreover, several studies connected Notch1 to stromal cells and inflammatory processes in TNBC by demonstrating Notch-mediated regulation of CXCL8 in this disease subtype (51, 56, 59–62). However, to date, our study provides the first mechanistic information on the regulation of CXCL8 by Notch1 in the tumor-stroma-inflammation network. Our data indicate that p65 is the prime regulator of CXCL8 expression and of Notch1 activation in this setting, and provide evidence to a molecular shift that takes place in TNBC:stroma co-cultures when they are stimulated by pro-inflammatory mediators such as TNFα.

Specifically, when tumor cells interacted with MSCs in “Contact” conditions in the absence of TNFα stimulation, they exchanged soluble materials and formed physical contacts that together led to CXCL8 induction, beyond the levels produced by each cell type alone and above the levels obtained in “Transwell” conditions in the absence of TNFα [(26); Supplementary Figure 2A1]. Our data indicate that without TNFα stimulation, CXCL8 produced in TNBC:MSC co-cultures was released exclusively by the tumor cells and resulted from basal p65 activation in the tumor cells. Moreover, in the absence of TNFα stimulation, basal p65 activation mainly in the tumor cells has induced Notch1 activation (Figures 9A1,C1), which then contributed to a contact-dependent induction of CXCL8 (Figure 7D).

However, the balance between the two cell types in their contribution to CXCL8 production was changed in the presence of TNFα stimulation, that led to further increase in CXCL8 release in the “Contact” co-cultures. The contacts between the tumor cells and the MSCs set the stage for the activities of TNFα which increased CXCL8 production, in a process that was entirely dependent on p65 activation (Figure 3B2). Now, in the presence of TNFα-induced signals, activation of p65 in the tumor cells has contributed much to CXCL8 release, and p65 activation in the MSCs provided its share as well. Also, in the context of TNFα stimulation, p65 activation that led to elevated Notch1 activation took place mainly in the tumor cells but also in the MSCs. Thus, a shift in regulatory pathways was induced by TNFα, eventually amplifying CXCL8 release to highest levels, when TNBC cells and MSCs interacted in the presence of TNFα stimulation.

Our observations also indicate that when the tumor cells and the stromal cells did not form physical contacts but exchanged soluble factors, TNFα stimulation gave rise to production of soluble factors; it is possible that these factors, together with TNFα itself have directly activated p65. Then, p65 which is well-known to be a strong inducer of CXCL8 transcription in other systems [e.g., (63)], has contributed to increased transcription of CXCL8 [as we have shown in our previous study (26)] in the tumor-stroma-inflammation network established herein.

In parallel, in the contact-dependent process, TNFα-driven activation of p65 - mainly in the tumor cells but also in the MSCs - has given rise to elevated Notch1 expression and activation. These effects could have been induced by processes of direct binding of p65 to Notch1 promoter, leading to increased NICD levels, as has been reported before (64, 65); in addition, Notch1 activation could have been induced by the TNFα-IKK pathway that was found to modify the function of molecules that participate in regulating Notch activation (66). The activation of Notch1 following TNFα stimulation has led to CXCL8 induction (Figure 7), possibly through the activity of elements that participate in Notch-induced transcription of target genes, such as p300 (67). Indeed, CXCL8 was found to be up-regulated in lung epithelial cells by p300 (68). Published studies indicate that the p300-mediated process of CXCL8 induction reflected interaction with the NF-κB pathway (68), further supporting our findings on p65-Notch1 cross-talk that regulates CXCL8 induction in our tumor-stroma-inflammation network in TNBC.

The overall outcome, therefore, was elevation in CXCL8 production that has reached its outmost levels only when TNBC cells interacted with stromal cells, and were stimulated by pro-inflammatory signals delivered by TNFα. Induction of CXCL8 in contact-dependent setting was driven partly by NF-κB-induced Notch1 activation. Moreover, since Notch1 activation following TNFα stimulation depended almost entirely on p65 activation, it is highly possible that Notch-mediated regulation of tumor cell migration and invasion was also induced by p65 activation, as result of TNFα activation.

To conclude, in our current study we have deciphered intricate mechanisms that control through p65 and Notch activation the interactions between TNBC cells and stromal cells in the context of the pro-inflammatory TME. We demonstrated key roles for Notch family members not only in inducing the expression of pro-metastatic chemokines such as CXCL8, but also in activating migratory and invasive capacities in the tumor cells upon their interactions with stromal cells in the presence of pro-inflammatory signals. In view of the fact that CXCL8 was revealed in our accompanying study as key regulator of many of the pro-metastatic activities of this network (26), the “take home message” of this study is that the interactions between the TNBC cells and the stromal cells have set the conditions that enabled TNFα to bring its effects to maximum, partly through p65-induced Notch1 activation that has led to CXCL8 induction, and consequently to other tumor-promoting activities.

Obviously, further elucidation of the roles of the different players of the TNBC-stroma-inflammation network is required; preferably, such investigations should be performed in syngeneic TNBC systems where the effects of Notch1 activation on the expression of the murine counterparts of CXCL8 could be investigated in the context of the *in vivo* TME. Here, it is important to indicate that analyses with TNBC cells that were manipulated to express lower Notch1 levels may be complicated by compensation mechanisms that lead to activation of other Notch receptors (as suggested by our preliminary results; Data not shown). In parallel, inhibitory modalities that target the Notch pathway could be used; however they are not specific for one particular Notch receptor and suffer from toxic side effects (39, 41).

Thus, we propose that Notch1-TNFα-CXCL8 studies in TNBC patients (e.g., patterns of expression and localization in the tumors) and the design of combined Notch + inflammation targeting modalities may be of great value when improved therapeutics for TNBC are looked for. Indeed, it is possible that by using Notch-targeting treatments together with inhibitors of pro-inflammatory mediators such as TNFα and CXCL8 receptors, the doses of Notch inhibitors could be reduced and their toxicities would be alleviated. This may be a realistic option, because inhibitors of TNFα for example have been successfully introduced to the clinical setting for the treatment of pro-inflammatory diseases (69, 70). Such combined modalities could be used in animal model systems and if they provide promising results, they could be considered as treatment options in TNBC patients. This newly-introduced approach may offer novel and promising treatments that would halt or limit disease progression in the most aggressive subtype of TNBC.

## Author Contributions

YL generated all data and was the leading scientist involved in all steps of research conduct. She was also extensively involved in manuscript preparation. SL contributed to establishment of research systems. TM established the CRISPR/Cas9 system. DM and LR-A participated in ELISA studies of Notch1 effects. DS provided insights to Notch-related aspects. SW participated in conception of this research in its initial stages. CK contributed to TCGA analyses. ME contributed to introduction of the Notch pathway to the study. AB-B, the principal investigator, was responsible for research conception, study design, data analysis and manuscript preparation.

### Conflict of Interest Statement

The authors declare that the research was conducted in the absence of any commercial or financial relationships that could be construed as a potential conflict of interest.
