# Supplementary material for: Notch-Mediated Tumor-Stroma-Inflammation Networks Promote Invasive Properties and CXCL8 Expression in Triple-Negative Breast Cancer
Source: Front Immunol. 2019 Apr 24;10:804. doi: 10.3389/fimmu.2019.00804 (PMC6492532; doi:10.3389/fimmu.2019.00804)
Supplement: Supplementary file 1 [file Data_Sheet_1.PDF]

Supplementary Figure 1

MDA-MB-231 + MSCs + TNFα

MDA-MB-231 + MSCs + TNFα + DAPT

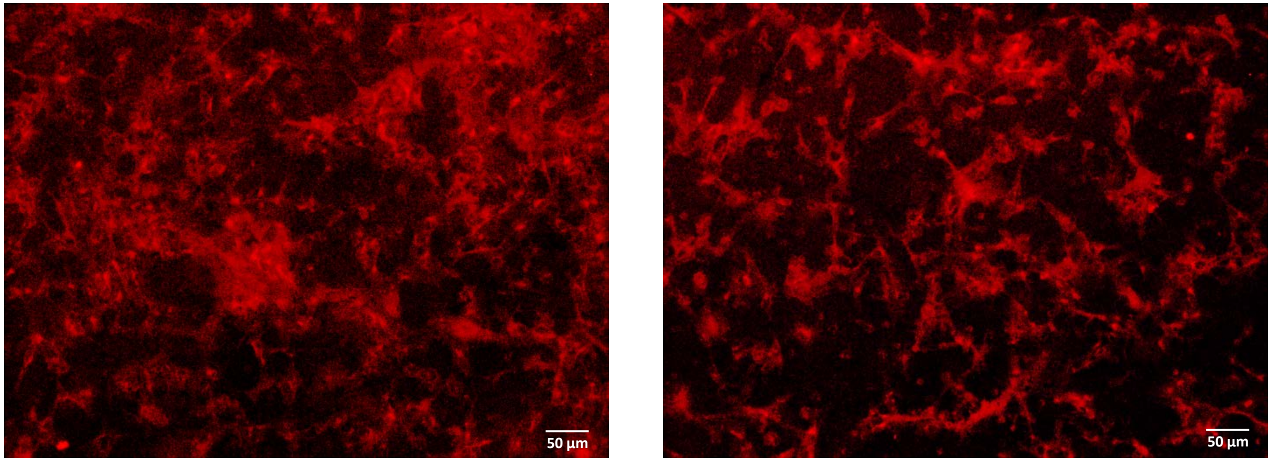

Supplementary Figure 1
mCherry signals, demonstrating the migration of mCherry-MDA-MB-231 cells following DAPT inhibition of TNFα-stimulated TNBC:stroma co-cultures
The Figure demonstrates the mCherry signals of the migrating cells presented in Figure 1A. The results are of a representative experiment of n=3 independent experiments, performed with MSCs of 2 different donors.

Supplementary Figure 2

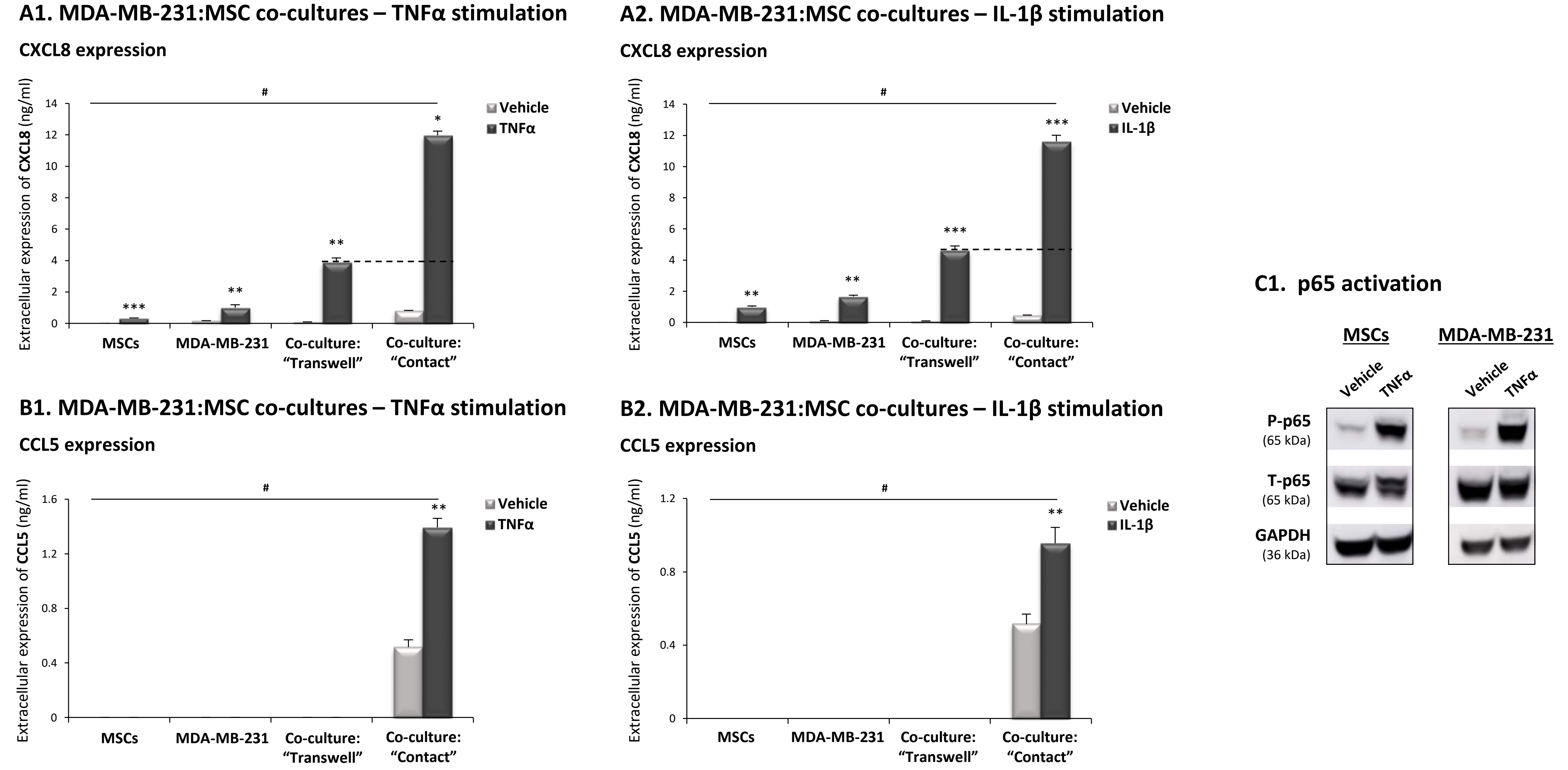

Supplementary Figure 2

Highest CXCL8 and CCL5 levels are released when TNBC cells and MSCs interact physically, in the presence of pro-inflammatory stimuli

The Figure demonstrates our findings on CXCL8 and CCL5 induction by TNFα and IL-1β stimulation (compared to vehicle control), in MSCs alone, MDA-MB-231 cells alone, and “Transwell”/”Contact” co-cultures. The data were presented in our previous study [26] and are included here again for readers’ convenience. Different representative experiments are shown in the two manuscripts. In brief, the cells were stimulated by TNFα (10 ng/ml), IL-1β (350 pg/ml) or vehicle for 7 hrs (Cytokine concentrations were selected based on preliminary titration analyses, as described in [26]). Co-cultures/cells were grown for additional 60 hrs in cytokine-free media, and CXCL8 levels **(A)** and CCL5 levels **(B)** in cell supernatants were determined by ELISA. ***$p \leq 0.001$, **$p \leq 0.01$, *$p < 0.05$ for differences between TNFα- or IL-1β-stimulated cells and vehicle-treated cells, within each group. #p values were ≤0.05, ≤0.01 or in most cases ≤0.001, in comparisons of “Contact” co-cultures with all other treatments, as well as in comparisons of “Transwell” co-cultures with all other treatments. The results are of a representative experiment of n>3 independent experiments, performed with MSCs of 3 different donors. **(C)** For readers’ convenience, the Figure demonstrates the activation of p65 in MDA-MB-231 cells and MSCs following 15 min of TNFα stimulation (10 ng/ml), as was presented in our accompanying study [26]. Different representative experiments are shown in the two manuscripts, n≥3 independent experiments, performed with MSCs of 2 different donors. WB quantification of the data of all experiments was presented in [26]. **Note:** The basal levels of p65 activation without TNFα activation are lower in this experiment than in Supplementary Figure 4C1 because in the latter the exposure time of the blots was elongated in order to see residual activation bands following p65 knock-out.

Supplementary Figure 3

A1. MDA-MB-231:<u>MSC</u> co-cultures – DAPT

CXCL8 expression

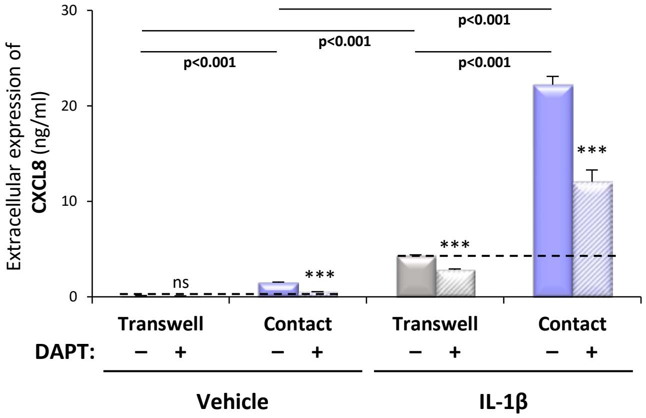

A2. MDA-MB-231:<u>CAF</u> co-cultures – DAPT

CXCL8 expression

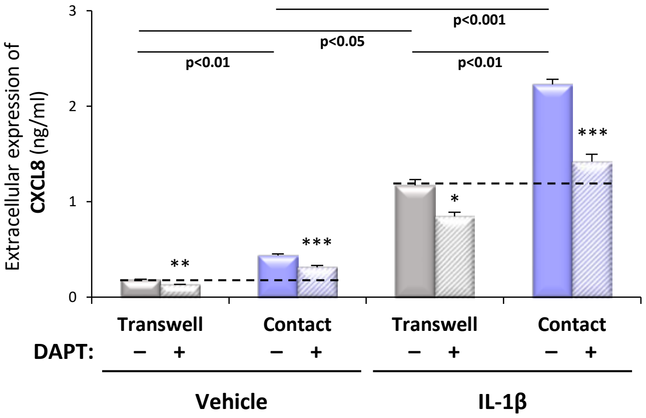

B1. MDA-MB-231:<u>MSC</u> co-cultures – DAPT

CCL5 expression

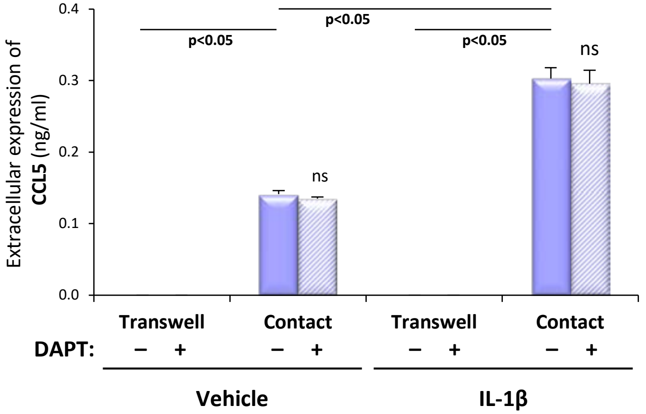

B2. MDA-MB-231:<u>CAF</u> co-cultures – DAPT

CCL5 expression

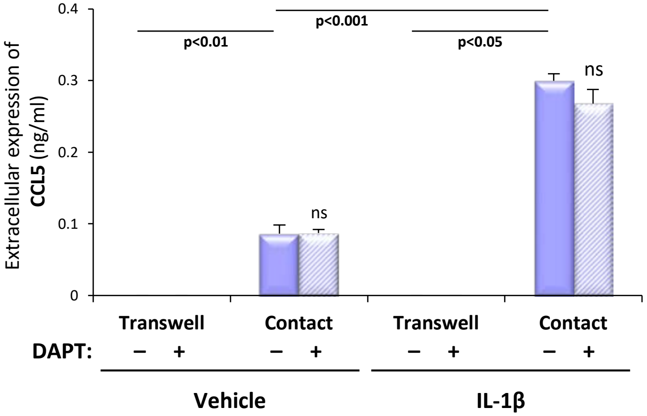

Supplementary Figure 3
DAPT inhibits the contact-dependent induction of CXCL8, but not of CCL5, in IL-1β-stimulated TNBC:stroma co-cultures
The Figure demonstrates the impacts of DAPT on IL-1β-stimulated co-cultures established by MDA-MB-231 cells and MSCs **(A1,B1)**, or by MDA-MB-231 cells and breast cancer patient-derived CAFs **(A2, B2)**, performed as described in Figure 2, with the exception that IL-1β (350 pg/ml) was the pro-inflammatory cytokine used herein (IL-1β concentration was selected based on preliminary titration analyses, as described in [26]). (A1) CXCL8 expression by MDA-MB-231:MSC co-cultures. (A2) CXCL8 expression by MDA-MB-231:CAF co-cultures. (B1) CCL5 expression by MDA-MB-231:MSC co-cultures. (B2) CCL5 expression by MDA-MB-231:CAF co-cultures. ***p<0.001, **p<0.01, *p<0.05, ns=non-significant for differences between DAPT-treated cells and control DMSO cells, within each group. The results are of a representative experiment of n>3 independent experiments, performed with MSCs of 2 different donors, and of n=3 independent experiments performed with patient-derived CAFs.

Supplementary Figure 4

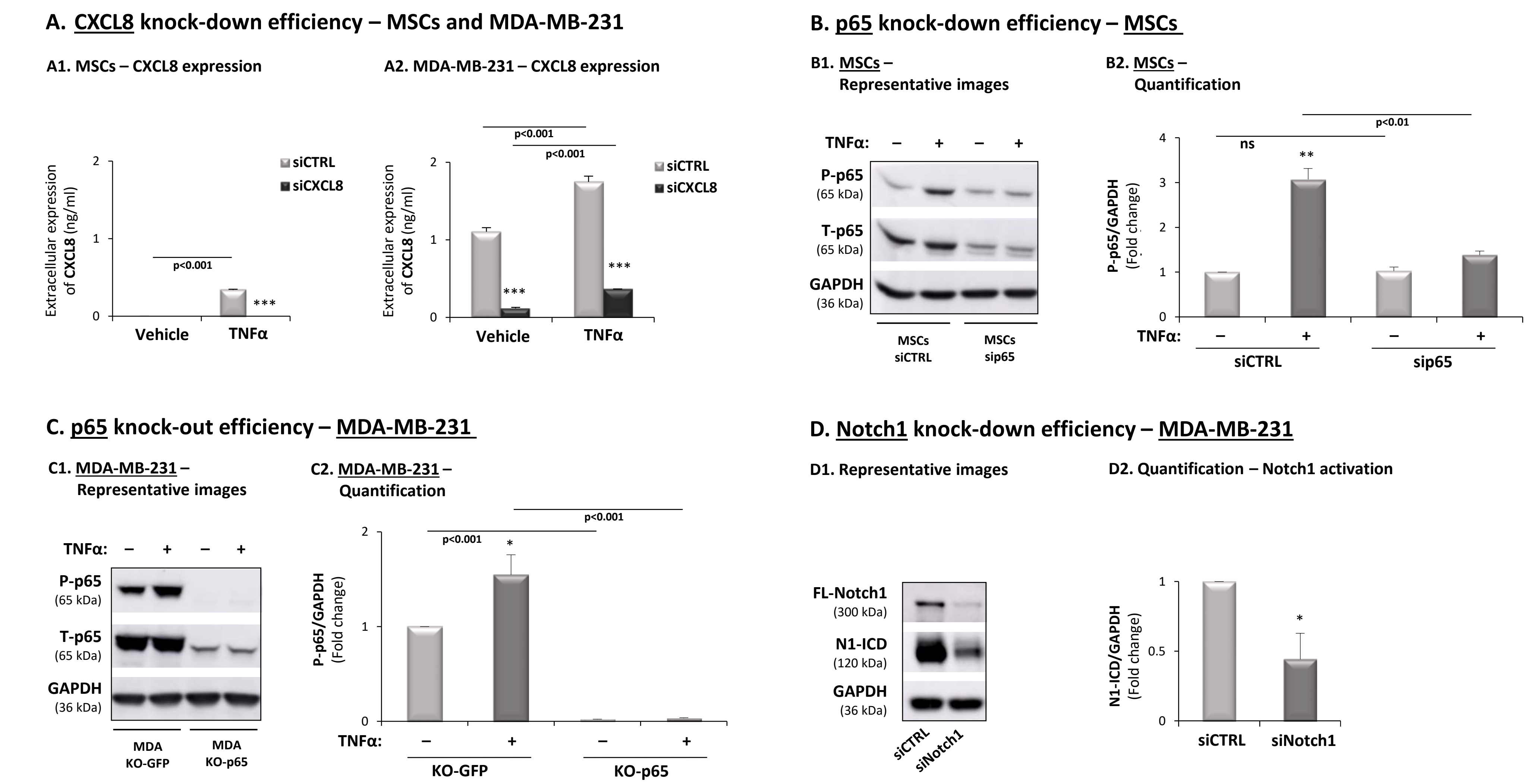

Supplementary Figure 4

Validations of knock-down/knock-out efficacies

(A) Efficiency of CXCL8 down-regulation was determined in MSCs (A1) and MDA-MB-231 cells (A2) that were transfected with CXCL8 siRNA or control siRNA (siCTRL). Extracellular CXCL8 levels in cell supernatants were determined by ELISA. **(B-C)** Efficiency of reduction in TNFα-stimulated p65 activation, induced in MSCs by p65 siRNA (B) and in MDA-MB-231 ("MDA") cells by CRISPR/Cas9 directed to p65 (C). The cells were stimulated by TNFα (10 ng/ml) and efficiency of p65 down-regulation was determined by WB. GAPDH was used as a loading control. (B1, C1) Representative experiments and (B2, C2) averages ± SD of p65 activation. Controls included control siRNA (siCTRL) in Part B, and sgRNA to GFP (KO-GFP) in Part C. **(D)** Efficiency of siRNA to Notch1 in reducing Notch1 expression and activation in MDA-MB-231 cells, compared to control siRNA (siCTRL), determined by WB. GAPDH was used as a loading control. (D1) Representative experiments and (D2) averages ± SD values of Notch1 activation. In all parts of the Figure, the results are representatives or averages of n≥3 independent experiments, performed with MSCs of 2-3 different donors ***$p<0.001$, **$p<0.01$, *$p<0.05$. ns=non-significant.

Supplementary Figure 5

A. siRNA-CXCL8 – Knock-down efficiency – IL-1β

A1. MSCs – CXCL8 expression

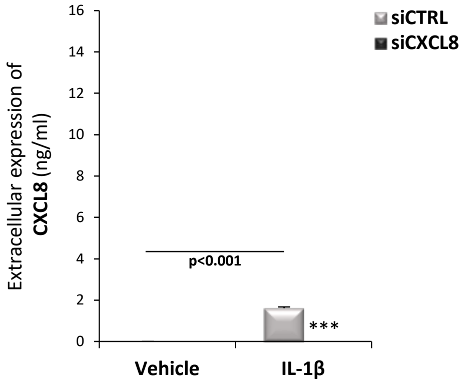

A2. MDA-MB-231 – CXCL8 expression

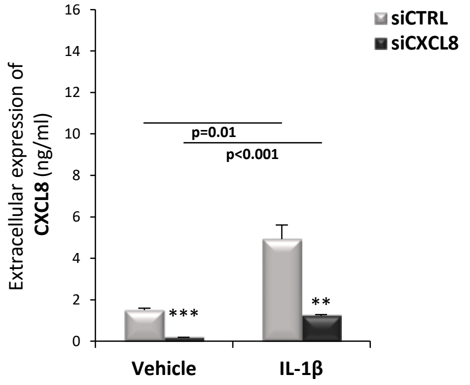

B. siRNA-CXCL8 – MDA-MB-231:MSC "Contact" co-cultures – IL-1β

B1. Vehicle-treated cells

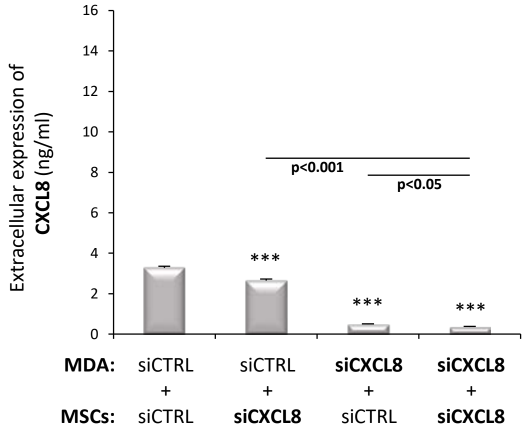

B2. IL-1β-stimulated cells

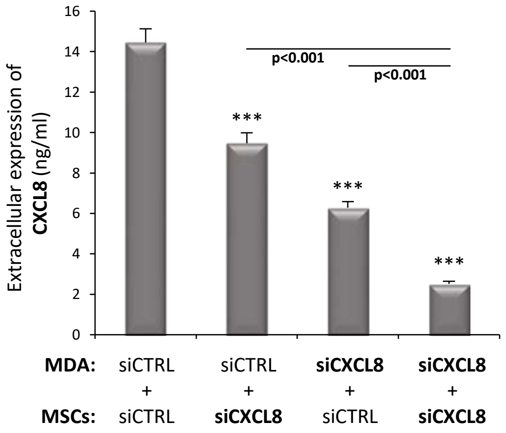

Supplementary Figure 5

In IL-1β-stimulated TNBC:MSC co-cultures, mainly TNBC cells but also MSCs, contribute to elevated levels of CXCL8

Studies with CXCL8 siRNA were performed as described in Figure 3A, with the exception that in the current figure the cells were stimulated by IL-1β (350 pg/ml). **(A)** Efficiency of CXCL8 down-regulation in MSCs (A1) and in MDA-MB-231 cells (A2). ***p<0.001, **p<0.01 for differences between siCXCL8-expressing cells and siCTRL-expressing cells. **(B)** The effects of CXCL8 siRNA on CXCL8 expression in vehicle-treated "Contact" co-cultures (B1) and IL-1β-stimulated co-cultures (B2). ***p<0.001 for differences between siCXCL8-expressing co-cultures and siCTRL-expressing co-cultures. The results are of a representative experiment of n=3 independent experiments, performed with MSCs of 2 different donors.

Supplementary Figure 6

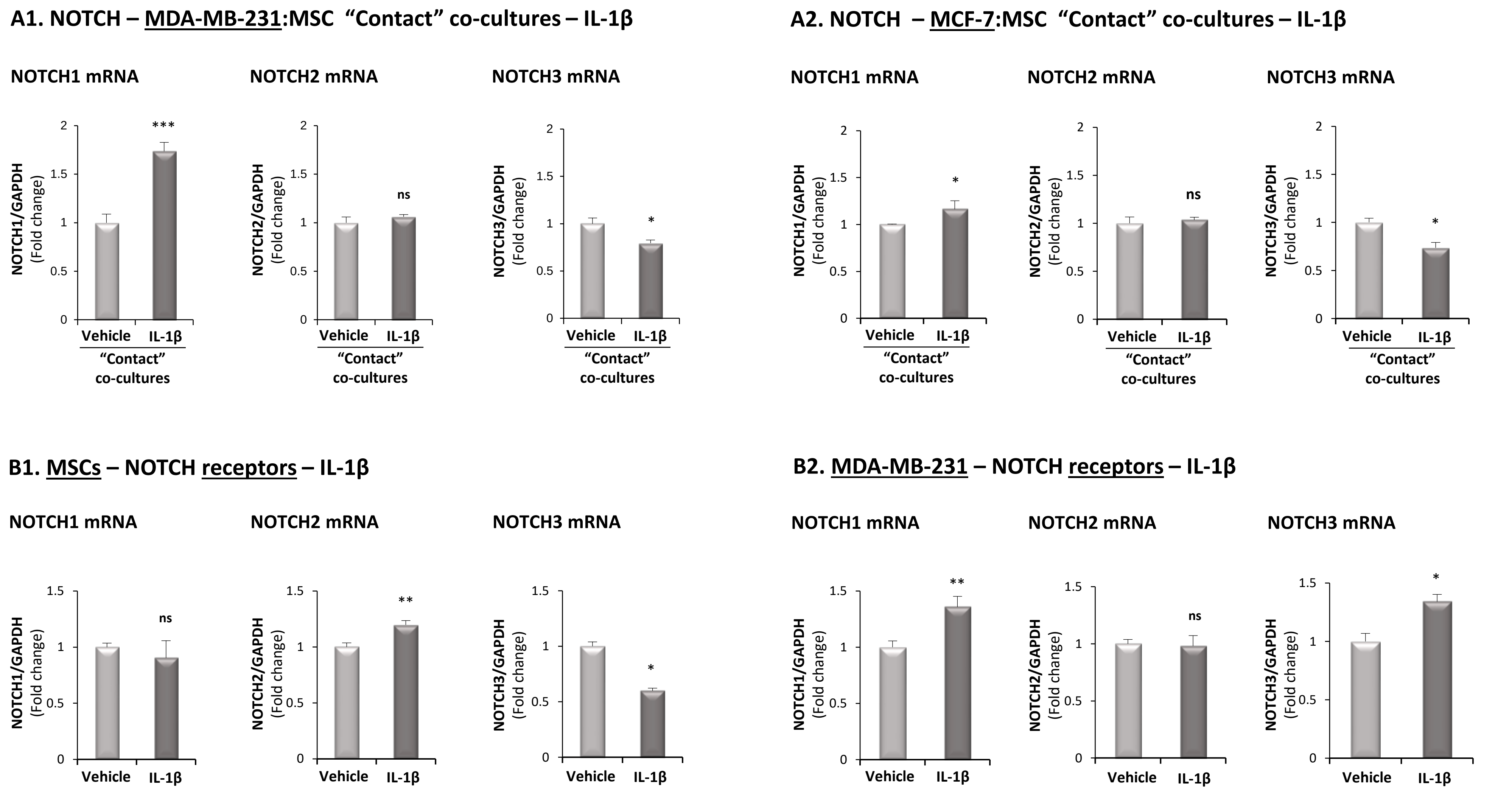

Supplementary Figure 6

Expression of Notch receptors following IL-1β stimulation in "Contact" cultures of MDA-MB-231:MSCs and MCF-7:MSCs, and in MSCs or MDA-MB-231 cells alone

(A) mRNA expression levels of NOTCH1, NOTCH2, and NOTCH3 were determined by qRT-PCR in MDA-MB-231:MSC (A1) or MCF-7:MSC (A2) "Contact" co-cultures stimulated by IL-1β (350 ng/ml) or vehicle control for 7 hrs. ***$p<0.001$, *$p<0.05$, ns=non-significant for differences between cytokine-stimulated and vehicle-treated co-cultures. **(B)** mRNA expression of NOTCH1, NOTCH2 and NOTCH3 in MSCs and in MDA-MB-231 cells, following stimulation by IL-1β (350 pg/ml) or vehicle control for 7 hrs. **$p<0.01$, *$p<0.05$, ns=non-significant for differences between cytokine-stimulated and vehicle-treated co-cultures/cells. In all parts of the Figure, the results are of a representative experiment of n≥3 independent experiments, performed with MSCs of 2 different donors.

Supplementary Figure 7

TCGA: Basal *vs.* Luminal-A

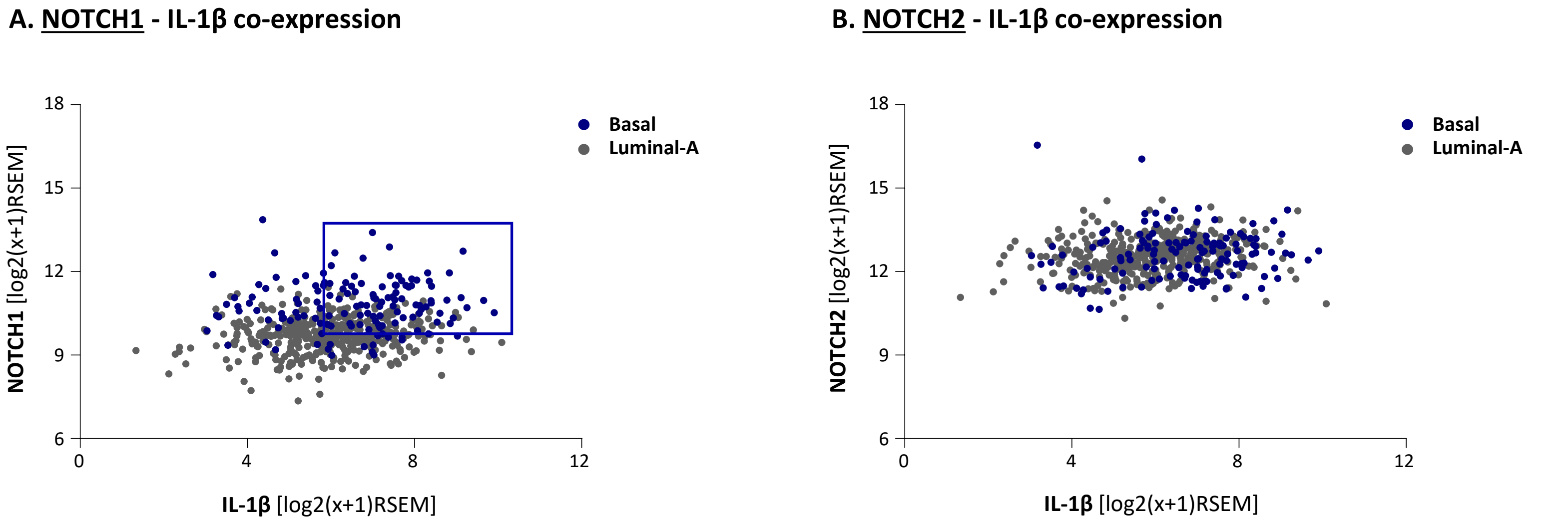

Supplementary Figure 7

The co-expression levels of NOTCH1 with IL-1β differentiate between basal patients and luminal-A patients

The Figure shows gene co-expression analyses of IL-1β performed using the TCGA breast cancer dataset, using the guidelines described in Figure 5. **(A)** NOTCH1 co-expression with IL-1β. **(B)** NOTCH2 co-expression with IL-1β. The blue rectangle illustrates the upwards-right shift observed in basal patients compared to luminal-A patients in NOTCH1 co-expression analyses, as described in Figure 6.

Supplementary Figure 8

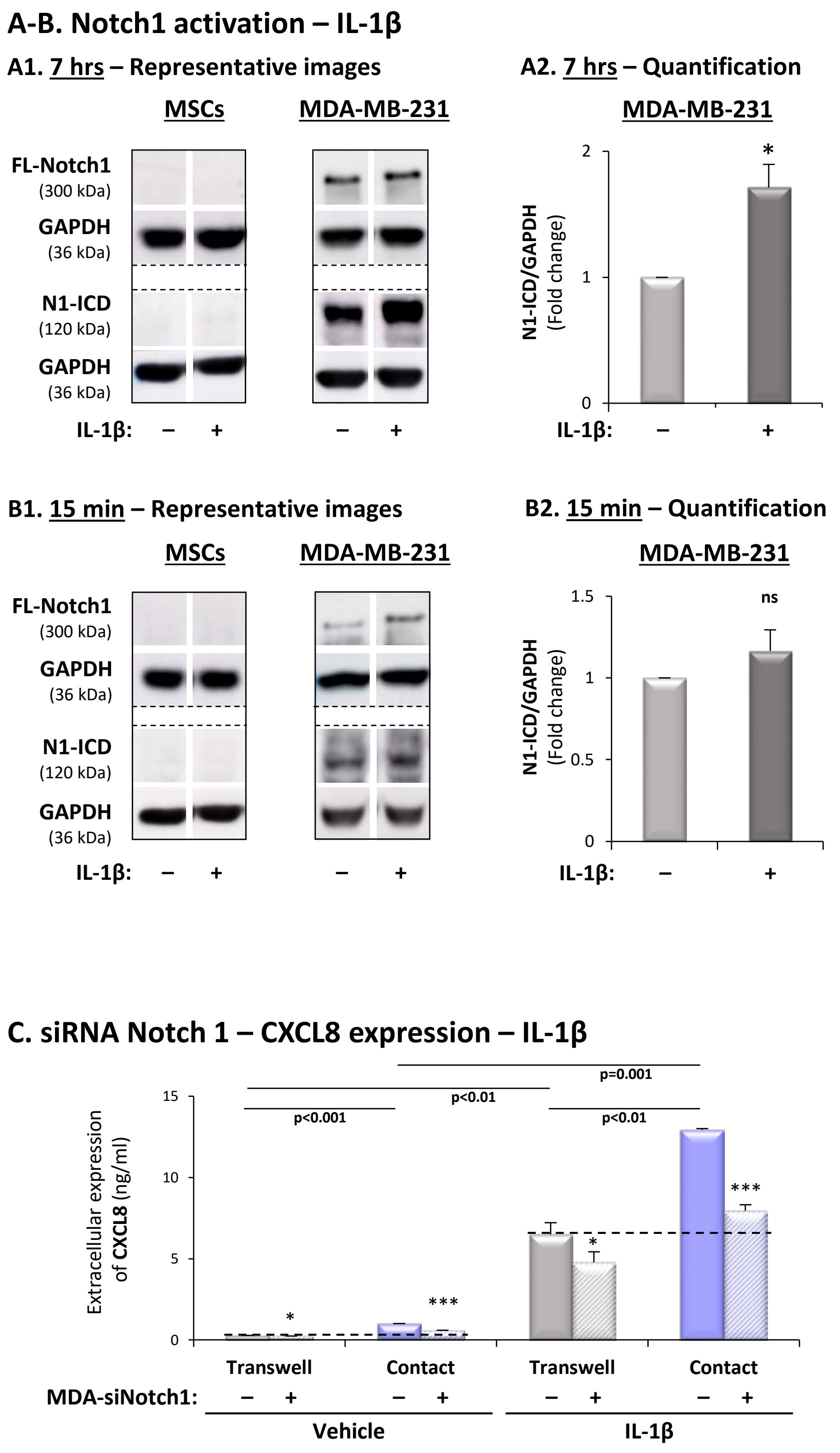

Supplementary Figure 8

Expression of Notch1 siRNA in TNBC cells inhibits the contact-dependent induction of CXCL8, in IL-1β-stimulated TNBC:MSC co-cultures

(A-B) Notch1 activation by IL-1β stimulation, following the guidelines described for Figure 7(A-B). (A1, B1) Representative experiments and (A2, B2) averages ± SD of Notch 1 activation in n=3 independent experiments, performed with MSCs of 2 different donors. *p<0.05, ns=non-significant. **(C)** Studies with Notch1 siRNA upon IL-1β stimulation, following the guidelines of Figure 7D. siRNA Notch1 efficiency in reducing Notch1 activation in MDA-MB-231 cells was demonstrated in Supplementary Figure 4D. ***p<0.001, *p<0.05 for differences between co-cultures with siNotch1-expressing MDA-MB-231 cells and co-cultures with siCTRL-expressing MDA-MB-231 cells. In all parts of the Figure, the results are of a representative experiment of n=3 independent experiments, performed with MSCs of 2 different donors.

Supplementary Figure 9

A. MSCs – NOTCH ligand mRNA – IL-1β

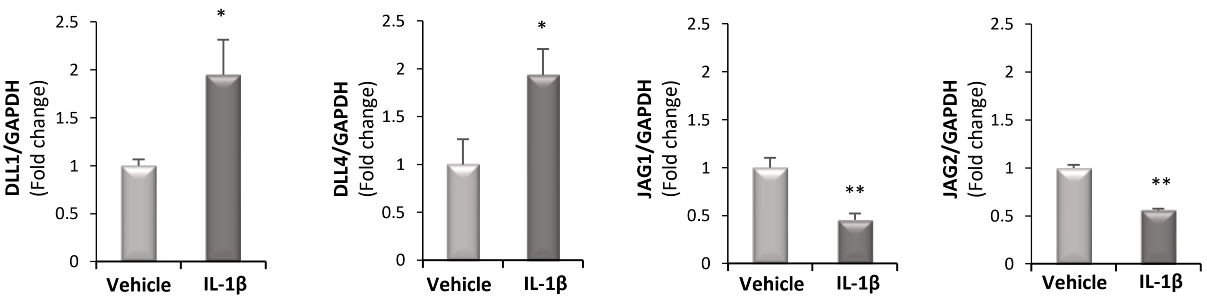

B. MDA-MB-231 – NOTCH ligand mRNA – IL-1β

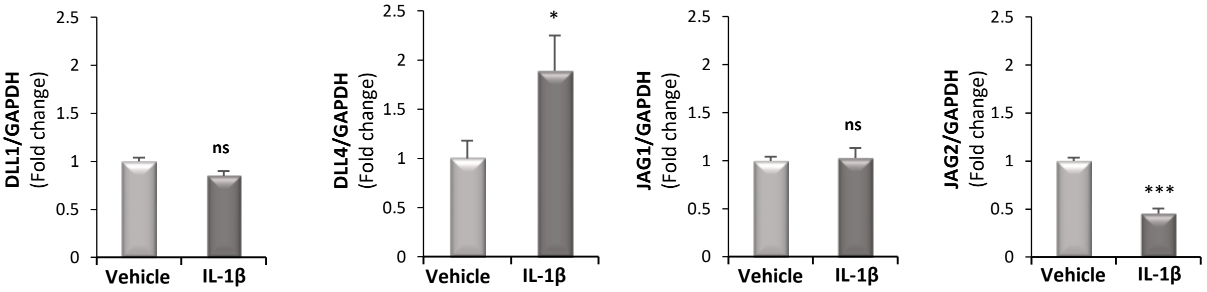

Supplementary Figure 9
Regulation of Notch ligand expression by IL-1β
The Figure demonstrates mRNA expression levels of Notch ligands, determined by qRT-PCR in MSCs **(A)** or in MDA-MB-231 cells **(B)** stimulated by IL-1β (350 pg/ml) or vehicle control for 7 hrs. ***$p<0.001$, **<0.01, *$p<0.05$, ns=non-significant for differences between cytokine-stimulated and vehicle-treated co-cultures. The results are of a representative experiment of $n\geq3$ independent experiments, performed with MSCs of 2 different donors.

Supplementary Table 1

Gene	Forward primer (5'-3')	Reverse primer (5'-3')
NOTCH1	ATGAGTTCCAGTGCGAGT	TGTAAGTGTTGGGTCCGT
NOTCH2	AGGAGGCGACCGAGAAGATG	CACAGGGTTCATAGCCATCTC
NOTCH3	GTGTGTGTCAATGGCTGGAC	GTGACACAGGAGGCCAGTCT
DLL1	TGCAACCCTGGCTGGAAA	AATCCATGCTGCTCATCACATC
DLL4	AGGCCTTGTTTTGTGACCAAG	GGTGCAGGTGTAGCTTCGCT
JAG1	ATCGTGCTGCCTTTCAGTTT	GGTCACGCGGATCTGATACT
JAG2	AGCTGGAACGAGACGAGTGT	TCTTGCCACCAAAGTCATCA
CXCL8	TTCTGCAGCTCTGTGTGAAG	CAGTGTGGTCCACTCTCAAT
GAPDH	CCACATCGCTCAGACACCAT	CAACAATATCCACTTTACCAGAGTTAA

Supplementary Table 1
Primers used in qRT-PCR analyses. For detailed description of qRT-PCR procedures, please see “Materials and methods”.

Supplementary Table 2

TNBC cells in "Contact" co-cultures with MSCs	Cytokine	# Exp.	DAPT-induced reduction in CXCL8 (%)
MDA-MB-468	TNFα	1	15.9*
		2	13.4*
		3	7.6
	IL-1β	1	18.1*
		2	13.7*
		3	6.7
BT-549	TNFα	1	19.1*
		2	3.9
		3	2.2
	IL-1β	1	21.4*
		2	13.3*
		3	0.3

Supplementary Table 2
The effects of DAPT on CXCL8 induction in MDA-MB-468:MSC and BT549:MSC co-cultures
"Contact" MDA-MB-468:MSC and "Contact" BT-549:MSC co-cultures were stimulated by TNFα (50 ng/ml and 25 ng/ml, respectively), IL-1β (500 pg/ml and 350 pg/ml, respectively) or vehicle control for 7 hrs. Then, exposure to DAPT (10 μM) or its vehicle (DMSO) was performed, and extracellular CXCL8 levels were determined by ELISA as described in Figure 2. The Table presents % reduction in CXCL8 levels due to DAPT treatment in n=3 independent experiments, performed with MSCs of 2 different donors. *$p<0.05$, for differences between DAPT- and DMSO-treated cells.
